# Do clones degenerate over time? Explaining the genetic variability of asexuals through population genetic models

**DOI:** 10.1186/1745-6150-6-17

**Published:** 2011-03-03

**Authors:** Karel Janko, Pavel Drozd, Jan Eisner

**Affiliations:** 1 Laboratory of Fish Genetics, Institute of Animal Physiology and Genetics, Academy of Sciences of the Czech Republic, Rumburská 89, 27721 Liběchov, Czech Republic; 2 Faculty of Science, University of Ostrava, Chittussiho 10, 710 00 Ostrava, Czech Republic; 3 Department of Theoretical Ecology, Institute of Entomology, Biology Centre of the Academy of Sciences of the Czech Republic, Branišovká 31, 37005 České Budějovice, Czech Republic

## Abstract

**Background:**

Quest for understanding the nature of mechanisms governing the life span of clonal organisms lasts for several decades. Phylogenetic evidence for recent origins of most clones is usually interpreted as proof that clones suffer from gradual age-dependent fitness decay (e.g. Muller's ratchet). However, we have shown that a neutral drift can also qualitatively explain the observed distribution of clonal ages. This finding was followed by several attempts to distinguish the effects of neutral and non-neutral processes. Most recently, Neiman et al. 2009 (Ann N Y Acad Sci.:1168:185-200.) reviewed the distribution of asexual lineage ages estimated from a diverse array of taxa and concluded that neutral processes alone may not explain the observed data. Moreover, the authors inferred that similar types of mechanisms determine maximum asexual lineage ages in all asexual taxa. In this paper we review recent methods for distinguishing the effects of neutral and non-neutral processes and point at methodological problems related with them.

**Results and Discussion:**

We found that contemporary analyses based on phylogenetic data are inadequate to provide any clear-cut answer about the nature and generality of processes affecting evolution of clones. As an alternative approach, we demonstrate that sequence variability in asexual populations is suitable to detect age-dependent selection against clonal lineages. We found that asexual taxa with relatively old clonal lineages are characterised by progressively stronger deviations from neutrality.

**Conclusions:**

Our results demonstrate that some type of age-dependent selection against clones is generally operational in asexual animals, which cover a wide taxonomic range spanning from flatworms to vertebrates. However, we also found a notable difference between the data distribution predicted by available models of sequence evolution and those observed in empirical data. These findings point at the possibility that processes affecting clonal evolution differ from those described in recent studies, suggesting that theoretical models of asexual populations must evolve to address this problem in detail.

**Reviewers:**

This article was reviewed by Isa Schön (nominated by John Logsdon), Arcady Mushegian and Timothy G. Barraclough (nominated by Laurence Hurst).

## Background

There is little disagreement that strict asexuality is an evolutionary dead-end in 'higher' organisms and that most asexual metazoans only form tips of the tree of life. Among the approximately 20 currently available hypotheses for the persistence of sex [1], those assuming time-dependent disadvantages of asexual reproduction (hereafter referred to as clonal decay) are widely accepted explanations for the apparent caducity of most natural clones and for the dominance of sex among Eukaryotes. Clonal decay may involve e.g. the gradual accumulation of deleterious mutations, low evolvability of clones, or exploitation by rapidly evolving parasites, which may also speed up the fitness decay in conjunction with other processes [2]. Empirical proofs of such mechanisms exist. For example, Paland and Lynch [3] and Neiman et al. [4] demonstrated higher rates of non-synonymous mutation accumulation in asexual strains of *Daphnia *water fleas and *Potamopyrgus *snails compared to their sexual counterparts. This indeed demonstrates inefficient purifying selection associated with clonal reproduction.

However, such results are also interpreted as evidence that increased accumulation of deleterious mutations is a prominent force that determines the ages of clones. We see this interpretation as problematic because strictly speaking, such studies document the proximate mechanisms underlying clonal decay but do not test whether selection really favours young clones and sexuals at the expense of ancient clones. Furthermore, such studies do not evaluate the generality of such processes because numerous studies failed to detect higher mutation or parasite loads in asexuals or prove comparable evolutionary plasticity to related sexual populations [[5-8], rev. in [9,10]]. Interestingly, Guex et al. [5] did not find lower fitness performance in hemiclonal lineages of water frogs at least 25 kya ancient compared to recently derived ones. Although Guex et al. [5] discuss putative parasexual processes that explain the similar performances of young and ancient asexual genomes, such findings may also suggest that at least in some cases, the ages of natural clonal genomes are not determined by clonal decay.

This emphasises the relevance of questioning how our assumptions about the consequences of asexual reproduction actually relate to real clonal lineages found in nature: do they really suffer higher extinction rates and shorter life spans resulting from time-dependent debilitation as predicted by theories? The answer to this question may seem obvious because available phylogenetic data suggest that asexual lineages are short lived. Detailed phylogeographic studies further demonstrate the restriction of ancient clones to more climatically stable regions where their sexual and younger asexual competitors are absent [11,12]. This is often interpreted as support for clonal decay and evidence suggesting that ancient clones may not persist in competition with young asexuals or sexual counterparts.

However, Janko et al. [13] demonstrated that such clonal distribution patterns do not deviate from neutral expectations and may not serve as a support for the clonal decay hypothesis. They also show that old clones disappear from an asexual population of finite size solely because of neutral drift even when clonal decay does not occur, provided that new clones regularly emerge from related sexual ancestors. Such neutral clonal turnover is analogous to the maintenance of neutral allelic variation under mutation-drift equilibrium. Thus, neutral clonal turnover is a special case of the clonal decay model, which, in addition to the stochastic component of clonal replacement, possesses a 'selection' component in which the fitness of clones decreases over time, e.g. due to deleterious mutation accumulation. From the qualitative viewpoint, both processes (i.e. clonal turnover and decay) predict the same patterns. Janko et al. [13] show that clonal diversity increases as the recruitment rate of new clones increases and that new clones replace existing ones more frequently, resulting in lower overall ages of clones. Thus, clonal diversity should generally be higher and clones should be less old in sympatric areas where asexuals coexist with their sexual ancestors because the probability of the origin of new clones is greater in sympatric areas than in allopatric asexual populations geographically isolated from sexual ancestors. Neutral clonal turnover also predicts that older clones are more widespread than younger clones on average, the same way as older alleles are more widespread than younger ones [14].

Although Janko et al. [13] show that neutral clonal turnover and clonal decay may not be disentangled by the qualitative comparisons of clonal distributions mentioned above, it is necessary to determine whether natural asexuals suffer from age-dependent fitness decay. To gain a quantitative insight into whether asexual taxa are shorter lived than sexual ones, Schwander and Crespi [15] compared terminal branch lengths in 14 published phylogenies, each comprising multiple asexual and sexual lineages. They found that current asexual taxa are not significantly younger than their sexual counterparts; this contrasts with the generally assumed role of asexuals as short-lived 'no-hopers'. However, as a potential caveat, the authors suggest that the definition of a species in sexual and asexual organisms must be equivalent in comparative studies. This may bias the results because the authors did not use any statistical analysis to detect which asexual lineages would be considered different species and hence be compared to sexual species (see [16]).

**Figure 1:**
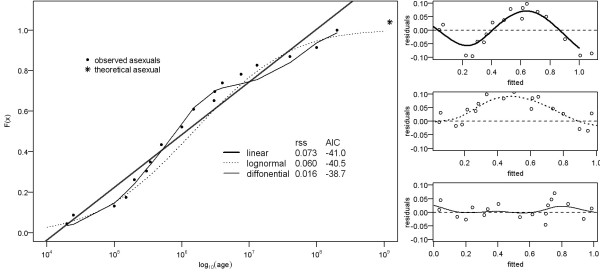
**Comparison of observed distribution with three types of theoretical distribution functions (loglinear cf. **[17]**, lognormal and 3^rd ^order multi-diffonential) on the left**. Residual sum of squares (rss) and Akaike information criterion (AIC) is calculated for each distribution and shown in legend. Fictive asexual with age 1.2 × 10^9 ^years is marked by asterix. Plots of residuals vs. fitted values (with trends estimated by smoothing) for above mentioned distributions are drawn on the right panel.

As an alternative approach, Neiman et al. [17] summarise the distribution of published age estimates of various asexual taxa, including three "scandalously" ancient asexuals (Oribatida, Bdelloida, and Darwinulidae) and draw two important conclusions. The authors suggest that the observed cumulative frequency distribution of asexual ages (Figure 1a) implies the existence of similar types of mechanisms determining maximum asexual lineage ages in all taxa. Neiman et al. [17] further show that asexual taxa with putatively high rates of clonal recruitment are not characterised by significantly lower age estimates than those characterised by low recruitment rates. This observation contrasts with the predictions of clonal turnover/decay models and led the authors to the conclusion that the ages of clones are not affected by the stochastic component (i.e. clonal recruitment rate) to a large extent.

The progress made by Neiman et al. [17] is important because their approach quantitatively addresses the generality and nature of underlying processes. Moreover, because they draw their conclusion only from the distribution of asexual taxa, they avoid possible bias due to the comparison of sexual and asexual taxa (see above). However, there are several weak points in their approach [17], and we suggest that their conclusions are open to alternative explanations and yet different approaches should be adopted to test the generality of processes affecting the distribution of clonal ages. In the following text we address three major issues. In part 1, we statistically revaluate the data of Neiman et al. [17] and show that the presented data distribution is unlikely to provide any strong conclusions regarding the universality of processes affecting clonal life spans. We subsequently show that the test of turnover model designed by Neiman et al. [17] is not appropriate because it violates the assumptions of clonal turnover model. In part 2, in order to propose an alternative approach, we use coalescent and individual-based simulations to demonstrate that rates of neutral clonal turnover and clonal decay mechanisms distort the mutation frequency spectra in multi-clonal asexual populations and that such effects are quantifiable by standard neutrality indices. In part 3, we derive several predictions to disentangle clonal decay and confounding demographic processes and use published sequences of clonal animals to test for deviations from neutrality. Finally, we discuss the implications for possible future research on the evolution of asexuality.

## Results and Discussion

### 1) Is the distribution of asexual ages informative about the nature and generality of underlying processes?

Neiman et al. [17] conclude that the life spans of clones are determined by general processes operating in a wide spectrum of studied taxa. Their conclusion is based on the fact that published age estimates of various asexual taxa have regular and nearly linear cumulative frequency on a log-transformed scale (Figure 1a), with no remarkable gaps separating the ancient taxa from the rest. However, because the authors inspected the frequency distribution only visually, they do not specify how large a gap in the distribution should be to reject its regularity. The low power of the visual method is obvious when a fictive asexual taxon that may be as ancient as sexual reproduction itself (i.e. 1.2 billion years [18]) is plotted to a published cumulative frequency distribution curve (Figure 1a). Such a log-scale distribution would still appear regular, and the fictive taxon would not appear as exceptionally old.

More importantly, the simple linear regression used by the authors, which we assume is produced by a uniform density distribution (UD) on a log scale, does not accurately fit the reported cumulative frequency; it results in systematically distributed residuals (Figure 1b) as well as relatively high residual sums of squares (Figure 1b). Other commonly used distribution models fit the empirical data comparably well or even better than the UD. We used R software [19] and the method of Šizling et al. [20] to fit two such distributions. The log-normal distribution, which is often used as a null hypothesis in macroecology [21], results in lower residual sums of squares (Figure 1a). An even better fit is achieved with application of multi-diffonential distribution (Figure 1a), which is a sum of several diffonential distributions. Because each diffonential term captures two counteracting processes [22,23], multidiffonential distribution is used to describe distributions generated by a mix of different underlying processes. It is notable that all three fitted distribution functions have comparable AIC scores [24] (Figure 1a); thus, it is difficult to decide which of them is better suited for the data.

This finding has important implications for future comparative studies of asexual organisms, because the age of an asexual lineage is always estimated from the distribution of nodes on sexual-asexual phylogenies. As pointed out by Birky and Barraclough [16], clusters of asexual individuals and their divergence from a sexual ancestor obey the stochastic rules of coalescence in an initial phase of asexual 'species' formation. Such clusters '...are not comparable to ecological or sexual species...' until they gain reciprocal monophyly from each other or from the sexual ancestor to form 'independent arenas for mutation, random drift, and selection' that are 'separated by long-lasting gaps with depths significantly greater than 2Ne generations' [16]. The coalescence-driven distribution of nodes on phylogenetic trees may dramatically differ from node distribution generated by 'interspecific' processes such as the birth-death process [25]. Therefore, estimates of clonal ages would differ among asexual taxa even if everything else was equal, depending on whether clones are affected by coalescence or the birth-death process. This suggests that both processes affect the age distribution reported by Neiman et al. [17] differently in young versus old taxa, which contrasts Neiman et al.'s conclusion about the generality of underlying process. It is important to note that even very simple distributions may fit the data generated by processes operating at dramatically different rates at different parts of the data distribution [22]. However, as stated by Preston [22], this fact may 'enable the insurance companies to set their premiums without running much risk--but it may have no physical meaning'.

Neiman et al. [17] further directly tested whether the ages of clones are determined by the rate at which new clones originate as predicted by the clonal turnover model. They suggest that if Janko et al. [13] are correct, then asexual taxa with faster rates of clonal recruitment should be characterised by higher numbers of independent clonal origins and lower age estimates. Neiman et al. [17] found no significant age differences between asexual taxa with monophyletic and polyphyletic origins of asexuality; therefore, they concluded that the model of neutral clonal turnover does not fit the data and that clonal ages are not determined by the stochastic component of clonal recruitment rate.

Although such quantitative evaluations of hypotheses explaining clonal age distributions are highly required, the test by Neiman et al. [17] is flawed. First, clonal turnover--an analogy of the mutation-drift equilibrium model in a finite population [26]--is only applicable to systems (we shall refer to them as 'asexual complexes' hereafter) where each new clone may enter a single arena for mutation, random drift, and selection with other clones (following Birky and Barraclough [16]). In contrast, Neiman et al. [17] violated this basic assumption of the clonal turnover model and pooled into a single analysis young asexual complexes undergoing the creation of new clones together with profoundly diversified asexual clades, which already meet the definition of a species and do not even have close sexual relatives (e.g. [16]).

Furthermore, the analysis of Neiman et al. [17] is based on a mixture of datasets produced from different sampling approaches and methods ranging from mtDNA sequencing to tissue grafts. We are concerned that comparative analyses based on such a heterogeneous dataset may be strongly biased. Indeed, the ability to detect the polyphyletic origin of clones depends not only on true clonal diversity but also on the variability of applied markers and the ancestral sexual population as well as sampling design (see [27,28] for review).

Lastly, splitting asexual taxa into single vs. multiple origins is not necessarily a good proxy for evaluating clonal recruitment rates because comparisons of clonal recruitment rates among unrelated asexual taxa are complicated (see Discussion in [13]). For example, according to the approach of Neiman et al. [17], Oribatid mites should be characterised by a higher recruitment rate of new clones, given the polyphyletic origin of asexuality in this group, compared to asexual *Ambystoma *salamanders, which are monophyletic. However, it is at the very least questionable whether several independent clonal origins inferred in the very old asexual taxon of Oribatid mites (asexual age ca. 200 Mya; see Table one in [17]) indicate higher origination rates than the monophyletic origin of *Ambystoma *asexuals that have a very short asexual history (25 kya). It follows that the interpretation of distribution of asexual ages is ambiguous, and other methods must be developed to address the processes determining the lifespans of clonal lineages and to rigorously test whether observed data deviate from neutrality.

### 2) Detecting age-dependent selection against clones from sequence data

It has been shown that clonal decay affects sequence variability in a predictable manner. Higgs and Woodcock [29] demonstrate that Muller's ratchet reduces the co-ancestry times of asexual individuals; Gordo et al. [30] show that it is also associated with a reduction of genetic diversity below classical neutral expectation. Because a whole asexual genome forms a single linkage group, such background selection [31] is likely to produce a considerable distortion in the neutral frequency spectrum toward an excess of rare variants in all loci including neutral ones [30]. Therefore, it should be possible to construct a comparative analysis of published phylogenetic data on asexual organisms and test whether they are systematically affected by clonal decay. The problem is that the above-mentioned studies [29,30] only assume intraclonal variability (i.e. consider only descendants of a single asexual founder of the whole clonal population; see [32]), and their predictions may not be directly applicable to data from natural sexual-asexual complexes. This is an important problem because distinguishing independent clones is very difficult from sequence data alone (e.g. [27]).

**Figure 2:**
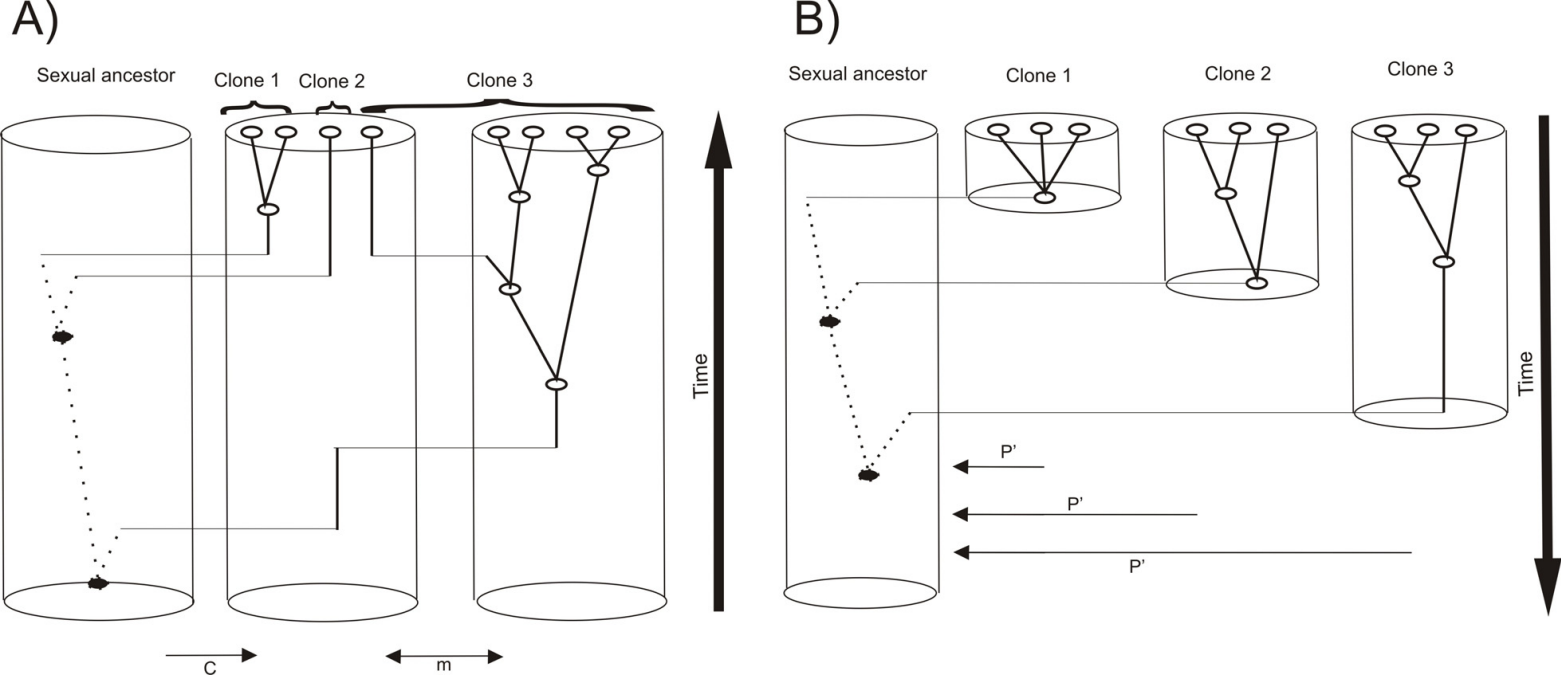
**A scheme of simulations**. A) A scheme of individual based simulation running forward in time: Asexual complex is composed of ancestral sexual population of size *N *and *d *asexual demes of size *N/d *each. New clones are born at rate *c *to the first asexual deme, which is connected to other demes by a migration rate *m *according to the finite linear stepping-stone model. B) A scheme of coalescent simulation running backward time: Asexual complex is composed of one or more ancestral sexual populations (in the latter case, they are connected by migration rate *m_c_*) and several clones with total population size *N_c _*distributed according to a broken stick model among clones. Looking backward in time, the clones either had a constant per generation probability *P' *of being founded by a single ancestor derived from a sexual progenitor (neutral model), or this probability raised per generation at rate [1 - (1 - f')^α'^)]. Solid lines denote the branches in the genealogy, which evolved during the asexual phase. Only those are used for the pruned dataset. Dotted lines represent the sexual phase of sequence evolution.

We used two methods to address the possibility of detecting traces of clonal turnover/decay from empirical data on polyclonal asexual complexes. First, we combined standard models of deleterious mutation accumulation [30,33,34] and extended them with additional parameters of clonal recruitment [13] together with routine population genetic models of structured populations. To do so, we used the individual-based model outlined by Janko et al. [13] and simulated the sequence diversity of geographically structured asexual complexes composed of ancestral sexual populations as well as asexual populations, which are constantly fed by new clones emerging from sexual ancestors. We assumed that each clone enters the same evolutionary arena for selection and drift (*sensu *[16]) and that equilibrium between the origination and extinction of clones may be established. We adopted a standardised method to model the stochastic accumulation of deleterious mutations [30] by assuming that the fitness of an individual is given by the relationship (1 - s)^k^, where *k *denotes the number of deleterious mutations and *s *denotes their selection coefficient. As a second approach, we constructed a coalescent simulation running backward in time and modelled clonal decay as a deterministic process where the probability of the extinction of any clone is a function of age and the rate of per-generation fitness loss is *f' *(see Methods and Figure 2). To study the effect of clonal decay on observed sequence variability, we overlaid a fixed number of segregating sites on resulting pedigrees and estimated values of Tajima's D (D) [35] and Fu and Li's D* (D*) [36]. These were then plotted as functions of the coefficients *s *and *f'*. We also studied the effect of demographic processes by varying the migration rates among both sexual and asexual demes.

It is very important to keep in mind that the sequence variability of asexual populations has two components: polymorphisms inherited from a sexual ancestor and mutations acquired after the formation of clones. Although processes solely operating among asexual individuals determine the variability of the latter component, the former one may be strongly affected by the population history of sexual ancestors. The dataset comprising solely mutations acquired after the switch to asexuality is designated 'pruned' hereafter, because it omits ancestral polymorphisms inherited from the sexual population; the dataset comprising all polymorphisms is referred to as the 'total' dataset. Consequently, we also evaluated the effect of clonal decay on both types of datasets (see Methods).

**Figure 3:**
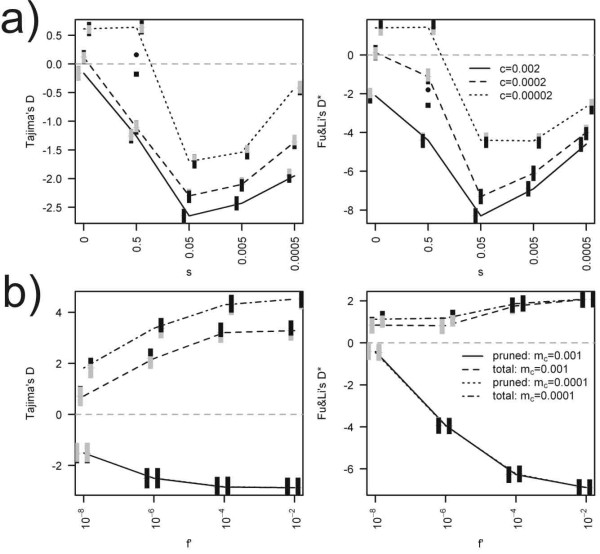
The effect of clonal decay on Tajima's D, Fu and Li's D*. Individual based simulation (upper panel) assumed a population of 500 individuals fed by new clones at rate c, deleterious mutations accumulating at rate 0.5, each with a selection coefficient s. Neutral model assumes s = 0. To demonstrate the effect of varying U, the black square and black dot symbols indicate the means values for U = 0.1 and U = 0.05, respectively. Selection coefficient s = 0.5 in both cases. Coalescent simulation (lower panel) assumed two sexual demes of a total size of N_c _= 25,000 individuals interconnected by migration rate m_c _and asexual population of N_c _= 25,000 individuals composed of 10 clonal lineages. Each clone had a constant probability P_c _of extinction per generation or it increased at rate (1 - (1 - f')^α'^). We sampled 5 individuals per deme/clone. Mutations in the neutral locus accumulated at rate 10^-8 ^in both cases. In the coalescent simulation, data are estimated according to mutation overlaid along the total pedigree (curves called 'Total') or along the branches evolved during the asexual phase of sequence evolution (curves called 'Pruned'). Black colour below or above the grey parts of bars indicates the proportions of cases where simulated values of D, D* were lower or higher than the 95% CI of the neutral expectation.

**Figure 4:**
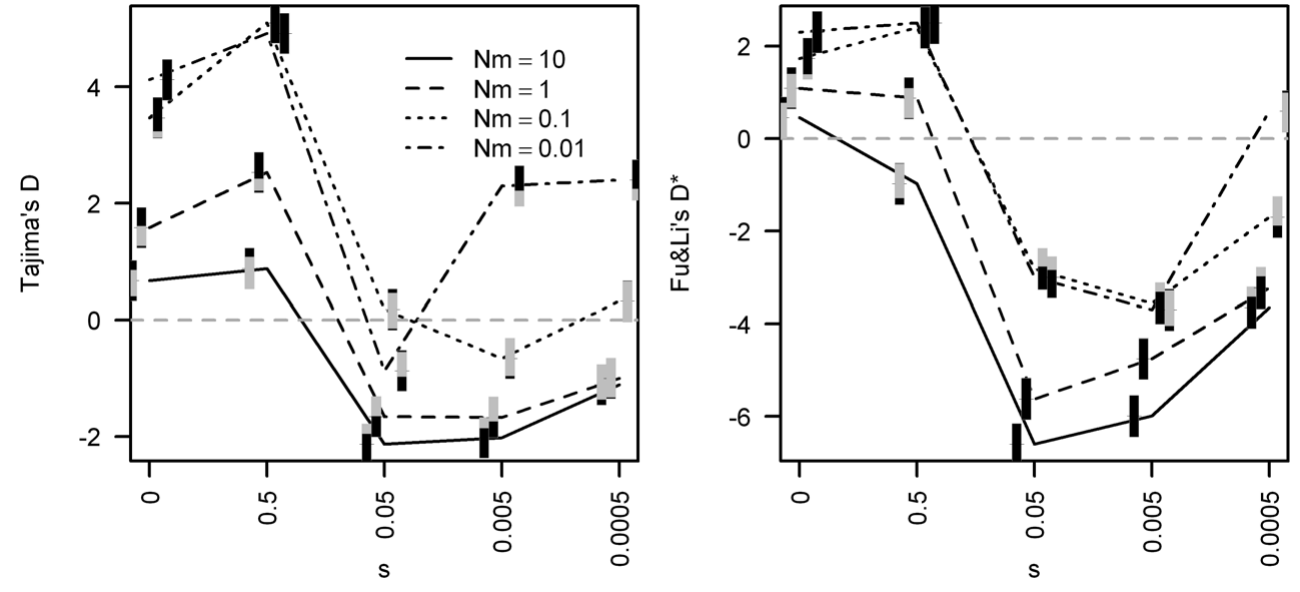
The effect of clonal decay on Tajima's D, Fu and Li's D* in a stepping-stone migration model of 5 populations each of N = 100 individuals. The first deme received an influx of new clones at rate 10^-3^. Deleterious mutations accumulated at rate 0.5 and mutation in the neutral locus at rate 10^-8^. Nm is the mean number of individuals immigrating into each population. Black colour below or above the grey parts of bars indicates the proportions of cases where simulated values of D, D* were lower or higher than the 95% CI of the neutral expectation.

We found that clonal decay distorts the mutation frequency spectra towards rare haplotypes in the pruned dataset, leading to negative values (often significant) of D and D* (Figures 3 and 4). This effect is insensitive to demographic processes in ancestral sexual populations. Under the model of deleterious mutation accumulation, this effect was correlated with the per-genome deleterious mutation rate U and was strongest for intermediate values of selection coefficients (Figure 3); this is in complete agreement with previous conclusions drawn from monoclonal populations [30]. Under the deterministic model of age-dependent loss of fitness (coalescent simulation), this effect was a monotone function of *f'*.

**Figure 5:**
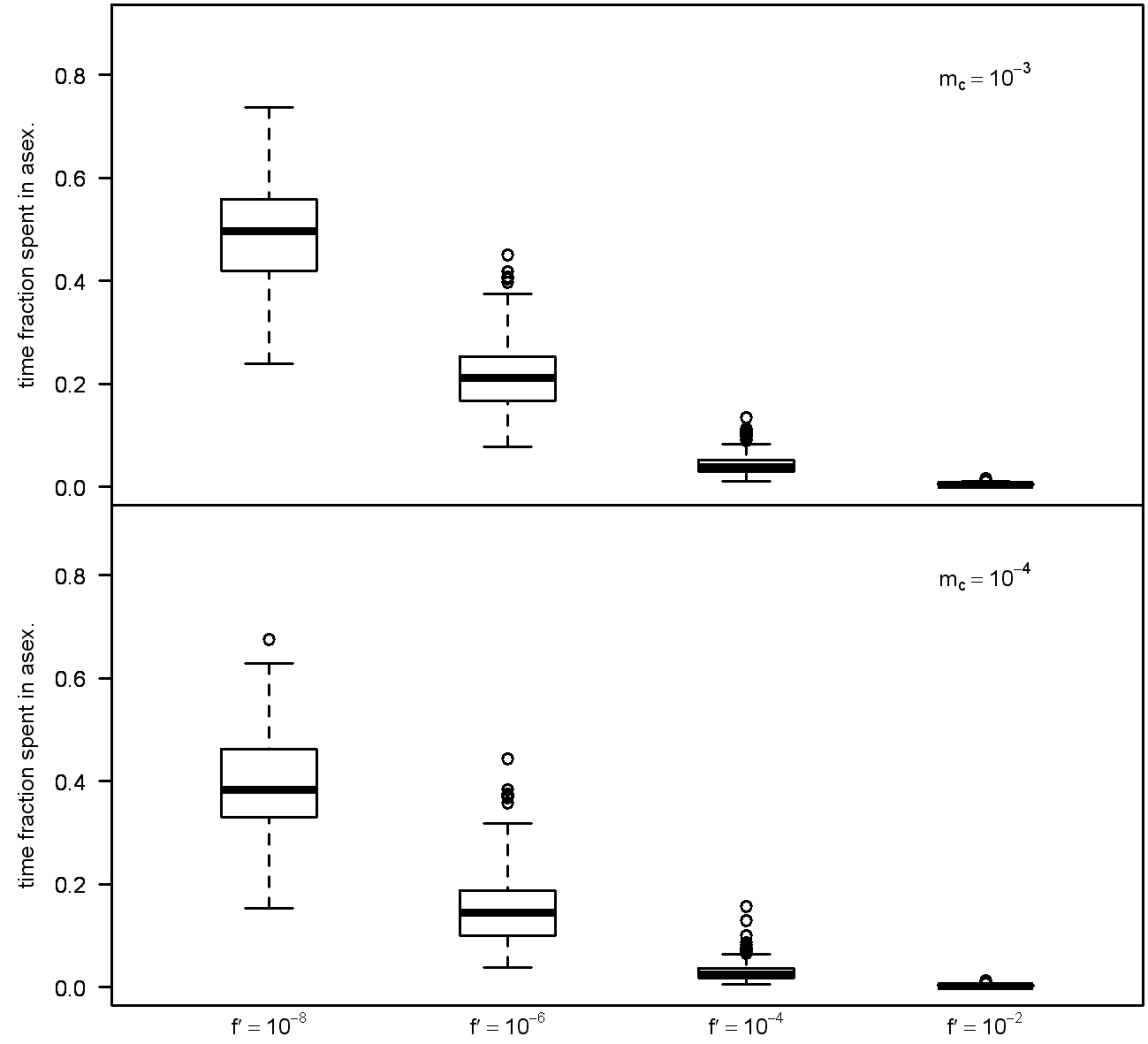
**Box plots demonstrate the fraction of time that simulated genealogies spend in asexual state as a function of age-dependent selection (f') and migration rate among sexual demes (m_c_)**. Note that as the selection pressure increases, the genealogies spend relatively longer time in sexual state. Note also that lower migration rate among sexual demes cause longer internal branches, which again result in shorter time spent in asexual state.

In contrast, values of neutrality indices estimated from the total dataset are strongly affected by processes in the sexual population. Figure 3b demonstrates that the more the sexual population is fragmented, the neutrality indices are more positive. We noticed that as the strength of clonal decay increases (and consequently, as the clones become shorter lived), the genealogies spend relatively longer times in the sexual phase (Figure 5) and the genetic footprints of processes affecting the sexual population become more prominent. Note that when the sexual population is fragmented, the neutrality indices tend to be more positive with increasing strength of clonal decay (Figure 3b).

Several neutral mechanisms distort mutation frequency spectra in a way similar to clonal decay. As shown by Janko et al. [13] and Combadao et al. [33], a pronounced population structure and low rates of clonal recruitment attenuate clonal decay and lead to longer-lived clones. In this study, we noticed that neutrality index values were negatively correlated with the recruitment rates of clones and migration rates among asexual demes (Figure 4). On the other hand, population fluctuations are known to turn the neutrality indices toward negative values [37]; hence, they may mimic the effect of clonal decay or increased clonal turnover.

### 3) Testing for clonal decay in real populations

#### 3.1 Definitions of model assumptions and predictions

Our findings suggest that if clonal turnover/decay affect the variability of clones, their sequences should bear traces of such processes. However, we must address several issues before attempting to detect footprints of selection against clones.

##### Dataset and model choices

As mentioned above, the clonal turnover/decay model is only applicable to asexual complexes, where each new clone may potentially replace any of the existing ones. Therefore, asexual taxa, which are composed of distinct asexual species (*sensu *Birky and Barraclough [16]) should be avoided. Our model further assumes unidirectional transitions from sex to asex. This assumption allows us to polarise substitutions on sexual-asexual pedigrees and to prune the dataset in order to focus on the polymorphisms that putatively evolved during the asexual phase only. On the other hand, this is also potentially limiting because it may lead to unpredictable behaviour in the case of massive asex → sex transitions. However, our assumption has a sound biological basis and is commonly used in previous papers addressing the minimum number of independent origins of asexuals (rev. in [17,38]). This paper is based on mtDNA datasets, and many asexual biotypes analysed here reproduce clonally, which theoretically prevents any gene flow to sexual species (e.g. [39,40]). If males (which may mate with sexual females) occasionally occur (e.g. [41,42]), they do not transfer mtDNA markers. Therefore, we consider the current model to be applicable for analysed dataset.

##### Pruning of sequences

polymorphisms inherited from sexual ancestors should be avoided in order to eliminate confounding effects from the sexual population. The effect of such 'noise' would be especially strong when variance inherited from sexual ancestors is higher than that acquired after the transition to asexuality. To avoid this, we propose two methods of pruning. First, we reconstructed artificial sequences of asexuals that do not take into account mutations that define phylogenetic branches connecting sexual individuals. In the second method, we eliminated all sites segregating in the sexual ancestor from a given dataset (see Methods). Both methods lead to very similar outcomes when analysing real datasets (see below).

##### Disentangling neutral turnover, clonal decay, and confounding demographic effects

Various neutral demographic processes may confound inferences based on the age distribution of clones and mutation frequency spectra. Unlike in sexually transmitted genomes, these processes may not be distinguished from selection using the standard multi-locus approach (e.g. [43]) because of the complete linkage of whole asexual genomes. Similarly, it would be possible to distinguish between simple clonal turnover and clonal decay if realistic estimates of relevant parameters such as the deleterious mutation rate or rate of clonal recruitment were available. However, their estimation is very difficult if not impossible. A clue to disentangle the processes that dominantly affect the lifespan of clones is that the intensity of confounding population processes and clonal turnover/decay are expected to be correlated with different traits. For example, the intensity of demographic fluctuations depends on latitude [44], whereas the rate of clonal turnover would positively correlate with the sympatric occurrence of a sexual ancestor [13]. Therefore, the identification of traits that significantly explain the distribution of empirical data may help to determine the predominant processes that affect the genetic build-up of asexual populations.

To test for systematic trends in the distributions of neutrality indices, we used available mtDNA sequences of asexual complexes and prepared four types of datasets: the total sequences of sexual and asexual individuals, respectively, as well as the two types of pruned sequences of asexual individuals (see Table 1 and Methods). We estimated the number of segregating sites (S) and three neutrality indices, D, D*, and D/Dmin, in each dataset. Furthermore, we evaluated the population size estimators (Θ_S_, Θ_π_) of sexual species from the total number of segregating sites S and mean pairwise sequence divergence (see Methods). Only taxa that could be approximated by clonal turnover/decay models were selected for the analysis (i.e. we did not include distinct species *sensu *Birky and Barraclough [16], see Methods). We propose several predictors of observed data distribution. At appropriate places, we list the reasons justifying the choice of such predictors. Fitting corresponding linear models test their strength and may identify dominant processes affecting asexual genomes.

**Table 1:** Summary of observed values

		Polymorphic sites				D			D/Dmin			D*		Age in Mya (gener./year)	Weight (length) [g (mm)]
				
**Ref**.	Species	Total	Prun1	Prun2	Total	Prun1	Prun2	Total	Prun1	Prun2	Total	Prun1	Prun2		
[42]	*Schmidtea polychroa*	42			-1.479			-0.482			-2.554				0.050 (8)
	*S. polychroa *asex. $,#,P	40	28	15	-0.275	-2.230	-1.754	-0.044	-0.818	-0.688	-0.388	-2.390	-2.080	0.75-1.5 (9)	

[64]	*Squalius pyrenaicus*	98			-1.400			-0.477			-1.538				150 (250)
	*T. alburnoides *$,#,P	130	110	67	-2.014	-2.670	-2.530	-0.681	-0.910	-0.876	-3.980	-6.190	-5.250	1.8-3.6 (1)	

[12]	*Potamopyrgus antipodarum*	22			0.930			0.421			-0.772				10 (90)
	*P. antipodarum *asex. $,P	36	29	21	-0.733	-1.560	-1.246	-0.280	-0.344	-0.231	-1.501	-2.660	-1.638	0.5 (2)	

[63]	*Ambystoma*	77			1.700			0.700			1.330				15 (90)
	*Ambystoma *asex. $,#,P	6			-0.629	-0.629	-1.440	-0.299	-0.299	-0.239	-0.503	-0.503	-0.380	LGM (1)	

[65]	*Daphnia pulex*	76			-1.953			-0.014			-2.982				0.002 (2)
	*D. pulex *asexuals	48	140	30	-1.878	-2.630	-2.361	-0.651	-0.900	-0.851	-3.582	-4.150	-3.480	0.172 (10)	

[66]	*Menetia greyi *SAS	52			-1.250			-0.310			-0.380				2 (50)
	*Menetia greyi *SAR	27			-1.220			-0.406			-1.200				
	RP parthenogens	12	12	12	1.233	1.233	1.233	0.526	0.526	0.526	0.703	0.703	0.703	NA	2 (50)
	WP parthenogens $	11	11	11	-2.172	-2.172	-2.172	-0.910	-0.910	-0.910	-2.827	-2.827	-2.827	NA	

[67]	*Campeloma limum*	84			0.484			0.283			0.335				5 (50)
	*C. limum *asexuals P	66	35	12	0.533	-1.350	-1.243	0.242	-0.493	-0.630	1.236	-1.940	-1.298	0.56 (1)	

[10]	*Cobitis elongatoides*	40			-1.370			-0.488			-1.700				15 (135)
	*C. elongatoides*-like asex. P	24	NA	13	-0.770	NA	-0.750	-0.288	NA	-0.306	-1.142	NA	-2.470	0.342 (1)	
	*Cobitis taenia*	45			-2.320			-0.782			-6.050				15 (135)
	*C. taenia*-like asex. P	7	NA	3	-0.560	NA	-0.530	-0.254	NA	-0.300	0.630	NA	-0.190	LGM (1)	
	*Cobitis *asex. pooled P	NA	19	NA	NA	-1.090	NA	NA	-0.424	NA	NA	-2.940	NA	0.342 (1)	15 (135)

[27]	*Phoxinus neogaeus*	1			NA			NA			NA				10 (90)
	*P. eos-neogaeus *asex.	5	4	4	0.062	0.263	0.263	0.031	0.129	0.149	0.980	0.886	0.886	LGM (1)	

[11]	*Timema poppensis*	27			-0.390			-0.060			-0.002				0.050 (50)
	4-2clade asex. $,#	9	6	2	-1.540	-1.920	-0.980	-0.670	-0.903	-0.635	-0.330	-2.360	-0.700	0.5 (1)	

[68]	*Bacillus rossius*	23			-1.070			-0.414			-0.470				0.050 (NA)
	*Bacillus *asex. P	10	10	6	-1.920	-1.920	-1.870	-0.829	-0.829	-0.880	-1.920	-1.920	-2.200	1.06 (1)	

[69]	*Aspidiotus*	34			-2.630			-0.970			-5.010				0.050 (10)
	*Aspidiotus *parthenogens	24			-1.110	-1.060	-1.270	-0.390	-0.415	-0.455	-1.170	-1.170	-1.400	1 (1)	

[70,9]	*Poecilia mexicana*	18													10 (90)
	*P. formosa *asex. $,#	10	10	6	-0.720	-0.720	-1.040	-0.305	-0.305	-0.500	-0.540	-0.550	-1.530	0.081 (3)	

[71]	*Warramaba*	105			0.240			0.160			-0.290				0.050 (NA)
	*Warramaba *parthenogens $,#	52			0.930	-0.360	-0.620	0.290	-0.114	-0.230	1.560	0.240	0.260	0.33 (1)	

[72]	***Leptynia***	23			-0.670			-0.210			-0.520				0.050 (70)
	*Leptynia *parthenogens $,#,P	15			0.780	-1.140	-1.400	-0.330	-0.530	-0.490	0.260	-1.020	-1.270	2.86 (1)	

For each complex we denote the variability as number of segregating sites in the total dataset, as well as after the first and the second type of pruning, respectively (Prun1, Prun2). Similarly, we note observed values of neutrality indices for each type of dataset. The sign $ denotes complexes with asexuals distributed (at least) partially beyond the distribution range of sexuals. The sign # denotes complexes, where asexuals extend to higher latitudes compared to sexual progenitors. 'P' denotes asexual complexes with predominant polyploid forms. The age-estimates of *Poecilia formosa *clonal lineages were based on the reanalysis of original data by Loewe and Lamatsch [9]. LGM stands for estimated origin of asexuals since the Last Glacial Maximum. SAS, SAR, RP and WP denote the different chromosomal races of Menetia lizards.

Predictor (1) clonal age: Clonal age should be a strong predictor of observed data distribution. Our model predicts a positive correlation between clonal age and neutrality indices; this is because as the rate of clonal turnover or the strength of Muller's ratchet increases, the average age of clones decreases [13], and measured neutrality indices tend to have negative values (Figure 3). In this analysis, we used published age estimates of clonal complexes as a predictor (Table 1). When the authors did not indicate a single age estimate but published an interval of plausible ages, mean values were used. Linear model (1) tests the dependence of neutrality indices on the age of asexuals.

**Figure 6:**
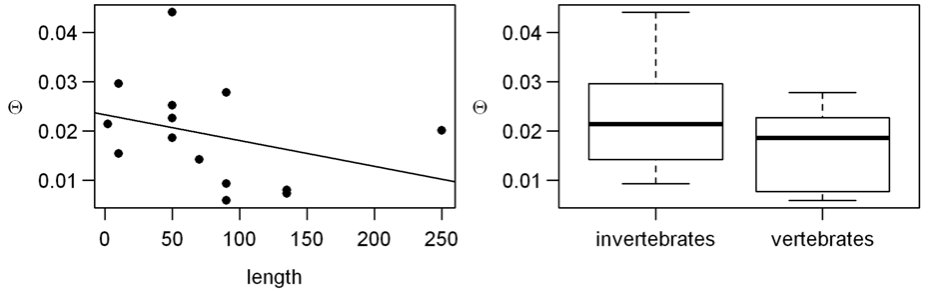
**Genetic polymorphism of sexual species expressed as Θ_S _in a function of body size (left) and the difference in Θ_S _between sexual vertebrates and invertebrates (right)**.

Predictor (2) population size: Increasing the size of an asexual population attenuates the rate at which Muller's ratchet clicks [1]; consequently, the values of D approach zero [30]. Therefore, asexual organisms with small population sizes should have lower neutrality indices due to the faster operation of Muller's ratchet. Unfortunately, we found no data on census population sizes and had to use some proxies. Bazin et al. [45] show that small organisms have greater genetic diversity and hence presumably larger population sizes than large ones. They also found lower DNA diversity in vertebrates compared to invertebrates, which presumably have larger populations. In accordance with Bazin et al. [45], we found some negative (though not significant) trends in the DNA diversity of sexual species relative to their body size; it also appears that vertebrates are less polymorphic compared to invertebrates (Figure 6). Therefore, we used body size measurements as a proxy for the population size of asexuals. Linear Models (2) and (3) measure the correlation between neutrality indices and body mass and length, respectively. We also tested whether estimated values of neutrality indices differ between asexual vertebrates and invertebrates (Linear Model (4)). In order to avoid the problem of substantial variability in published estimates of body mass and length, we pooled studied taxa into appropriate weight/length categories (Table 1).

Predictor (3) geographical isolation from sex: According to clonal turnover/decay models (rev. in [13], see Introduction), clones living in (partial) isolation from their sexual ancestors are less likely to be replaced by new clones. Consequently, asexual taxa fully sympatric with sexual ancestors should be characterised by generally younger clones and lower index values compared to parapatric/allopatric complexes (Figure 3 and 4). We split the complexes (Table 1) into two groups on the basis of whether asexual lineages occurred in (at least partial) isolation from the sexual progenitor. Table 1 reveals that geographical isolation from sexual ancestors indeed affects the age composition of clones in a predictable manner: the average age of allopatric complexes is 1.13 Mya vs. 0.37 Mya in sympatric ones (one-sided *t*-test, t = -2.011, df = 16, *p *= 0.03). Linear Model (5) tests whether geographical isolation also affects the genetic variability of asexual populations.

**Figure 7:**
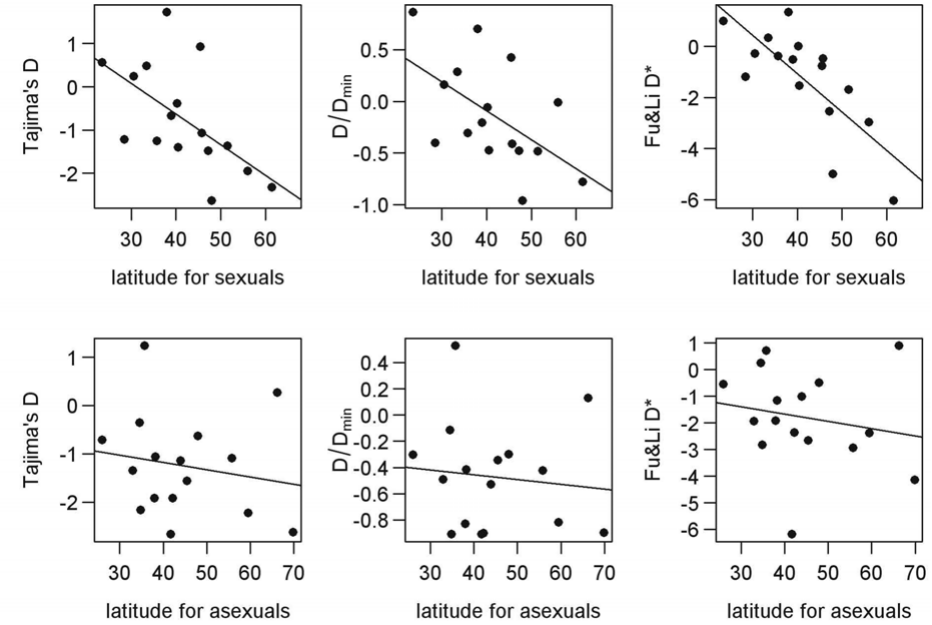
Upper panel: Regression of D, D/Dmin and D* values for datasets of parental sexual species against the highest latitude of their distribution; Lower panel: Regression of D, D/Dmin and D* values for datasets of asexual complexes against the highest latitude of their distribution. Sequences are pruned according to the first pruning method.

Predictor (4) extreme habitats and latitude: According to Schwander and Crespi [14], the recent age of asexuals can be explained by their tendency to occupy extreme habitats in higher altitudes/latitudes, which are more prone to founder-flush events than the distribution ranges of sexual progenitors. Strong correlations exist between latitudinal distribution and the intensity of population oscillations; furthermore, populations of boreal animals are more genetically homogeneous than long-term fragmented and stable populations of tropical species (e.g. [44]). In agreement with these facts, the values of neutrality indices measured in sexual species (Table 1) are significantly correlated with their latitudinal distribution (D: R^2 ^= 0.43, *p *= 0.008; D/Dmin: R^2 ^= 0.34, *p *= 0.02, D*: R^2 ^= 0.69, *p *< 0.01; Figure 7a). We have shown that fragmentation of asexual populations attenuates the turnover of clones [13] and drives the neutrality indices toward positive values (Figure 4), whereas population fluctuations tend toward the opposite. Therefore, we expect that if demographic oscillations significantly affect clonal populations, this effect should increase with increasing latitude. Linear Model (6) tests the correlation between observed data and the highest latitude of complex distribution.

Predictor (5) data pruning: We predict that if data pruning systematically affects observed index values, then the distribution of neutrality indices would be correlated with the amount of original genetic variability deleted during the pruning process. To address this issue, Linear Model (7) tests for the correlations between observed data and the ratio of the number of segregating sites after pruning data to that before pruning (S_pruned_/S_total_).

Predictor (6) sample sizes: The efficiency of pruning relies on the ability to distinguish between 'frozen' and post-formational polymorphisms. This may be affected by the fact that the closest sexual ancestor of any clone might have remained unsampled. Such bias should be strong if the sexual ancestor is poorly sampled, while good sampling should eliminate it. Linear Model (8) tests whether the data distribution is systematically affected by the sample size of sexual species.

Predictor (7) ploidy: Polyploidy may have significant effect on mutation rates and efficiency of purifying selection [46]. This may in turn affect the rate at which fitness of clones deteriorates over time. Therefore, we have assorted the asexual complexes into groups where diploid or polyploid biotypes predominate (Table 1). Linear Model (9) tests whether polyploidy has some effect on the genetic variability of asexuals.

The series of these linear models *sensu *Farraway [47] (E(D) = β_0 _+ β_1 _* X; where X stands for a given explanatory variable), including the null model assuming no dependence of data (*β_1 _= 0*), were ranked according to their predictive power using the Akaike information criterion with the second order bias correction term (AICc; [48]) in the R programming platform. We also evaluated the evidence ratio for each model [49], which is a relative measure of how much more likely any model from our set is compared to the null model. We also calculated regression statistics for each model (i.e. R^2^, coefficient of determination) and tested their significance by ANOVA. We did not construct more complex models because of the risk of overfitting due to the low number of available data on asexual complexes [49].

### 3.2 Identifying dominant processes affecting the genetic diversity of asexuals

We observed very low R^2 ^values (0.003 and 0.053) and evidence ratios while analysing the total sequence variability (data not shown). Analyses based on both pruned datasets indicate very low evidence ratios in Linear Models (2-9), but consistently demonstrate strong negative correlations between neutrality indices and the ages of clones (Linear Model (1); R^2 ^= 0.36-0.60; Table 2).

**Table 2:** Summary of Linear Model ranking according to AICc:

Pruning 1									
**Index**	**LinearModel**	**Rank**	**Explanatory variable X**	**K**	**logL**	**AICc**	**Emin,j**	**β_1_**	**R^2^**

D									
	null	3	---	2	-0.356	5.634	1.000	0.264	
	1	1	log(age in generations)	3	9.108	-9.816	2264.677	-0.449	**0.601
	2	7	log(body_mass)	3	0.124	7.753	0.347	0.087	0.058
	3	10	body_length	3	-0.656	9.713	0.130	-0.001	0.006
	4	4	vertebrate or invertebrate(boolean)	3	0.773	6.459	0.567	0.747	0.131
	5	9	isolation_from_sex (boolean)	3	-0.250	8.682	0.218	-0.573	0.071
	6	8	latitude of asexuals (in degrees)	3	-0.100	8.201	0.277	-0.015	0.031
	7	6	(Spruned/Stotal)	3	0.281	7.439	0.406	-0.524	0.076
	8	5	number_of_sampled_sexuals	3	0.390	7.402	0.413	-0.001	0.004
	9	2	Diploid or Polyploid (boolean)	3	2.630	3.418	2.7	-0.501	0.082
D/Dmin									
	null	2	---	2	14.631	-24.339	1.000	0.103	
	1	1	log(age in generations)	3	21.432	-34.464	158.001	-0.157	**0.518
	2	4	log(body_mass)	3	15.407	-22.814	0.467	0.043	0.092
	3	10	body_length	3	12.607	-16.814	0.023	0.000	0.001
	4	3	vertebrate or invertebrate(boolean)	3	15.661	-23.321	0.768	0.281	0.121
	5	9	isolation_from_sex (boolean)	3	13.867	-19.552	0.091	-0.239	0.080
	6	7	latitude of asexuals (in degrees)	3	14.726	-21.453	0.236	-0.004	0.012
	7	5	(Spruned/Stotal)	3	14.980	-21.960	0.304	-0.154	0.043
	8	8	number_of_sampled_sexuals	3	14.454	-20.727	0.164	0.000	0.001
	9	6	Diploid or Polyploid (boolean)	3	15.127	-21.588	0.231	-0.162	0.060
D*									
	null	5	---	2	-9.189	23.301	1.000	0.459	
	1	1	log(age in generations)	3	-4.728	17.856	15.221	-0.737	*0.360
	2	10	log(body_mass)	3	-9.163	26.327	0.220	-0.035	0.003
	3	3	body_length	3	-7.061	22.522	1.477	-0.012	0.166
	4	9	vertebrate or invertebrate(boolean)	3	-9.133	26.266	0.294	0.298	0.007
	5	8	isolation_from_sex (boolean)	3	-8.916	26.013	0.258	-0.469	0.016
	6	7	latitude of asexuals (in degrees)	3	-8.912	25.824	0.283	-0.027	0.034
	7	6	(Spruned/Stotal)	3	-8.556	25.111	0.405	-0.909	0.076
	8	4	number_of_sampled_sexuals	3	-7.371	22.925	1.207	-0.006	0.063
	9	2	Diploid or Polyploid (boolean)	3	-6.892	22.451	1.451	-1.258	0.115

**Pruning 2**									

**Index**	**LinearModel**	**Rank**	**Explanatory variable**	**K**	**logL**	**AICc**	**Emin,j**	**β**	**R^2^**
D									
	null	2	---	2	1.640	1.576	1.000	0.227	
	1	1	log(age in generations)	3	10.767	-13.352	1744.804	-0.321	**0.5002
	2	6	log(body_mass)	3	2.089	3.668	0.351	0.073	0.0514
	3	10	body_length	3	0.988	6.205	0.099	-0.001	0.0053
	4	3	vertebrate or invertebrate(boolean)	3	2.442	2.963	0.543	0.546	0.0899
	5	8	isolation_from_sex (boolean)	3	2.002	3.996	0.298	-0.642	0.1103
	6	9	latitude of asexuals (in degrees)	3	1.658	4.530	0.228	-0.004	0.0021
	7	7	(Spruned/Stotal)	3	1.982	3.883	0.316	0.747	0.0394
	8	4	number_of_sampled_sexuals	3	2.420	3.160	0.453	0.001	0.0036
	9	5	Diploid or Polyploid (boolean)	3	2.623	3.419	0.399	-0.501	0.0801
D/Dmin									
	null	2	---	2	16.780	-28.703	1.000	0.093	
	1	1	log(age in generations)	3	24.009	-39.836	261.606	-0.129	**0.4851
	2	4	log(body_mass)	3	17.526	-27.206	0.473	0.038	0.0840
	3	10	body_length	3	14.668	-21.155	0.023	0.000	0.0000
	4	3	vertebrate or invertebrate(boolean)	3	17.853	-27.861	0.462	0.257	0.1187
	5	8	isolation_from_sex (boolean)	3	15.723	-23.447	0.072	-0.178	0.0506
	6	6	latitude of asexuals (in degrees)	3	16.805	-25.764	0.230	0.002	0.0030
	7	5	(Spruned/Stotal)	3	17.467	-27.088	0.446	0.431	0.0777
	8	7	number_of_sampled_sexuals	3	16.689	-25.377	0.190	0.000	0.0007
	9	9	Diploid or Polyploid (boolean)	3	15.127	-21.588	0.045	-1.623	0.0608
D*									
	null	2	---	2	-7.032	18.922	1.000	0.378	
	1	1	log(age in generations)	3	-1.506	11.193	47.659	-0.634	**0.4321
	2	8	log(body_mass)	3	-7.032	21.910	0.224	0.001	0.0000
	3	3	body_length	3	-5.562	19.305	0.825	-0.008	0.1078
	4	6	vertebrate or invertebrate(boolean)	3	-7.011	21.868	0.276	0.151	0.0024
	5	5	isolation_from_sex (boolean)	3	-6.513	21.026	0.349	-0.858	0.0710
	6	7	latitude of asexuals (in degrees)	3	-7.024	21.895	0.226	-0.004	0.0009
	7	9	(Spruned/Stotal)	3	-7.032	21.911	0.224	0.001	0.0000
	8	4	number_of_sampled_sexuals	3	-5.754	19.507	0.746	0.002	0.0054
	9	10	Diploid or Polyploid (boolean)	3	-6.892	22.451	0.141	-1.258	0.1470

Part 1 relates to results based on the first method of data pruning and part two relates to the second pruning method (see the text for details). For each Linear Model of the formula E(D) = β_0 _+ β_1 _* X, we note its reference number as in the text, its final ranking, we describe its explanatory variable X, total number of parameters (K), log Likelihood (logL), AICc, Evidence Ratio (Emin,j) expressing how much more likely is given model compared to the null model, regression coefficient β_1 _and R^2^. * and ** in column R^2 ^stands for significance at α = 0.05 and 0.01.

The observed results allow us to draw some general conclusions about nature of processes affecting the genetic variability of asexuals. It is evident that both pruned datasets bear traces of the same processes that affect asexual gene pools. Methodological artefacts of data pruning or inefficient sampling apparently do not largely bias our results because Linear Models (7) and (8) have very poor fit.

**Figure 8:**
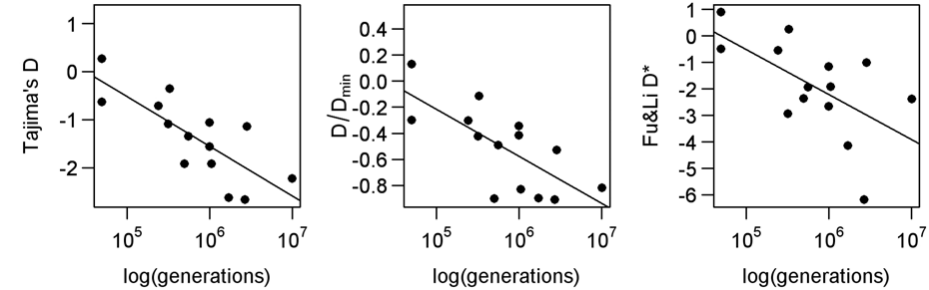
Regression of D, D/Dmin and D* values for datasets of asexual complexes against the logarithm of ages in generations. Sequences are pruned according to the first pruning method.

Furthermore, there are two lines of evidence suggesting that demographic history does not significantly interfere with the examined datasets of asexuals. First, if demographic processes play prominent roles in shaping asexual genetic variability, we would expect a positive correlation between neutrality indices and clonal ages (see above for explanation), which was not the case (Figure 8). Second, asexual datasets show no sensitivity to latitude, while the diversity of their sexual ancestors confirmed a strong effect of latitude (see above and Figure 7). This suggests that dominant processes affecting asexuals' variability differ from those operating in sexual species. It is indeed possible that the population sizes of individual clones undergo considerable fluctuations. However, our analysis accounts for not only intraclonal variability, which may be sensitive to such population sweeps, but also for polymorphisms along internodes joining independent clones to their common sexual ancestor. Variability in these positions reflects processes operating on the whole asexual population and is not largely affected by demographic fluctuations of individual clones.

The shape of the phylogenetic relationships among asexual individuals may not be explained by stochastic turnover alone, since clonal turnover predicts positive correlations between clonal age and neutrality indices (see above); meanwhile, we observed negative correlations (Linear Model (1); Figure 8 and Table 2). Instead, it seems that clonal age has some deterministic effect on the genetic build-up of asexual populations, because older asexual complexes are characterised by stronger deviations from neutrality than younger ones. However, the underlying mechanism may not be approximated by simple age-dependent fitness loss and it also differs from the commonly assumed model of Muller's ratchet (e.g. [30,34]) since both such processes also predict a positive correlation between clonal age and neutrality indices. Furthermore, we observed that body size has no effect, although we admit that it represents only a very rough proxy for population size estimates.

Applied models for sequence evolution predict an opposite trend than that observed from empirical data. Nevertheless, there are two reasons to believe that our approach warrants further attention. First, our analysis identified the age of clonal complex as the most important variable explaining observed data distribution. Indeed, just this parameter has played a central role in the research of asexuality for decades. Second, turnover/decay models apparently fail to explain the genetic variability of asexual complexes, but they correctly predict geographic and age distribution patterns of clones in qualitative (e.g. [13]) and even quantitative ways (see the definition of the Predictor (4) in the section above). We tend to interpretation that the applied population model pinpoints some relevant features of clonal evolution, but it misses some important parameters. This situation is not uncommon in the history of attempts to tractate natural systems by mathematical or statistical models. For example, very different estimates of gene flow may result from models assuming migration-drift equilibrium [50], non-equilibrium models evaluating the additional parameter of the split-time between demes [51], or even models taking into account the time-dependent decrease in migration rates [52]. However, to some extent, all of these models provide valuable insights into the dynamics of studied populations.

### 3.3 Implications for further studies of the evolution of asexuality

Our findings have several important implications. We relaxed the assumption of monoclonality inherent in previous studies (e.g. [30]) and demonstrated the suitability of sequence data for addressing selective forces even in asexual complexes comprised of multiple independent clonal lineages. By simultaneously comparing multiple taxa, we demonstrated that different processes affect the genetic variability of asexual and sexual populations, which is a promising extension to previous studies on individual clonal complexes (e.g. [3]). The present results also suggest some deterministic role of clonal age on fitness. However, our study demonstrates that a combination of routinely available population genetic models with previously published models for sequence evolution in asexuals [13,30,33,34] does not adequately address all major processes operating in real populations. This highlights the need to ameliorate the conceptual framework for understanding the dynamics of asexuality before any conclusive interpretations are made. In following two paragraphs, we shall discuss aspects that may be of interest in building more explicit population models of secondary evolution of asexuality.

The present model is based on the assumption of equilibrium between the recruitment and extinction of clones. Weak (if any) signals of clonal decay observed in some 'younger' complexes may result from the fact that asexuality originated only recently in such cases and equilibrium had not yet been reached. Janko et al. [13] discuss a potential analogy with migration-drift equilibrium and recent analytical progress in phylogeography and population genetics suggests that such departures from equilibrium may be analytically or at least numerically tractable (e.g. [53,54]). The ability to discriminate between equilibrium vs. non-equilibrium asexual complexes depends on the design of particular population models as well as initial conditions. However, to our knowledge, there is no consensus on how secondary asexuality originates. For example, there may be relatively continuous influx of new clones since the first appearance of asexuality (as in our model); it is also possible that they may originate in bursts (e.g. when two hybridising species meet under some favourable change of conditions) with little or no origins of clones between such events. Any model dealing with a non-equilibrial state should take the initial conditions into account.

On the other hand, if we consider studied asexual complexes as dynamic systems with on-going clonal turnover (see [55]), the terms 'young' or 'recent' would refer to complexes where asexuality is in fact 'ancient' and clones never had a chance to substantially diverge from sexuals. In such a case, the increasing signal of clonal decay in older complexes would suggest that some initial time is necessary for the selection to differentiate between 'recent' and 'ancient' clones. It is possible that periods of stasis are followed by rather abrupt changes in environmental conditions. A certain period of time would then be needed for the environment to change substantially from the conditions to which the clone was optimally adapted at the time of its origin. Only asexual complexes with clones old enough to 'remember' such a change in conditions would be subject to this form of selection. Such older complexes may be also characterised by stronger deviations from neutrality because older clones would have passed through more demographic fluctuations than younger asexual complexes. Alternatively, the increasing signal of selection in 'older complexes' could be explained in terms of the theory of mutational meltdown [56]. This theory predicts inefficient selection during the initial phase of clonal evolution as a result of low variance in the numbers of mutations among individuals. However, there is no existing analytical solution for polyclonal complexes. It is also possible that epistasis plays some role and that the detrimental effects of mutations may be widely enhanced in older lineages, which have accumulated a greater mutation load. Unfortunately, theoretical work only concerns monoclonal populations and suggests that the effect of epistasis varies according to the distribution of mutational effects (e.g. [57] and citations therein). Moreover, contemporary empirical data largely differ in their estimated magnitude and even in the sign of the epistasis (rev. in [58]).

### 3.4 Conclusions

Although theoretical studies stress the importance of long-term disadvantages of asexuality for the maintenance of sex, contemporary empirical evidence for such processes is ambiguous (see the Introduction and section 2). The importance of this paper to the discussion about the evolution of asexuality is fivefold: a) it demonstrates the possibility to detect clonal decay from sequence data in polyclonal populations; b) we have found that genetic diversity of clonal organisms is under control of different mechanisms than that of sexuals; c) deviations from neutrality are proportional to clonal ages in studied complexes, which corroborates the hypothesis of long-term disadvantage of asexuality; d) on the other hand, nearly neutral values of indices observed in some 'younger' complexes point at an under-appreciated possibility that the appearance and vanishing of clones may obey stochastic rules of drift in situation, where clones are not old enough to be debilitated by fitness deterioration; finally e) we have shown that contemporary models are insufficient to fully describe the nature of mechanisms affecting long-term fitness of clones, suggesting the need to identify the relevant parameters for more explicit models.

## Methods

### Simulation Models

In order to quantify the effect of background selection on sequence variability in clonal complexes we conducted two kinds of simulations.

The first one was an individual-based simulation (IBS) as described in Janko et al. [13]. Briefly, we simulated haploid clonal population of size *N*, which was split into *d *demes connected by migration rate *m *per generation according to the finite linear stepping-stone model. New clones were born at rate *c *to the first deme only. Clonal individuals received a Poisson-distributed number (*U*) of new deleterious mutations per generation, such that the fitness of an individual carrying *k *mutations was given by *(1 - s)^k ^*[30] where *s *stands for selection coefficient of each mutation. In each simulation run we recorded the pedigree of *z *individuals randomly drawn from each sampled deme. A whole asexual pedigree coalesced in a single sexual node, which was also the direct ancestor of all newly arisen clonal individuals and the most recent common ancestor of the whole pedigree (MRCA; Figure 2a).

In the second case (Figure 2b), we performed coalescent simulation running backward in time, which allows simulations of much higher population sizes. We modified the original metapopulation model of Pannell [59] assuming several demes. The first *d_C _*demes harboured sexual population of the size *N_C_*, while the remaining *L_C _*demes were asexual and corresponded to independent clonal lineages. (To be more precise, each clone occupied one asexual deme, see Figure 2b). Population sizes of all demes were distributed according to a broken stick model and summed up to a total of *N_C _*asexual haploid individuals. There was constant per-generation migration rate *m_C _*connecting the sexual demes according to a standard finite-island model. We reconstructed a coalescence history of z_C_*L_C _asexual individuals (where *z_C _*denotes the number of samples per clone), until the whole pedigree coalesced in a single sexual MRCA. Looking backward in time, the clones either had a constant per generation probability *P' *of being founded by a single ancestor derived from a sexual progenitor (neutral model), or this probability raised per generation at rate *[1 *- *(1 - f')^a'^]*, where *a' *denotes the age of clonal lineage and *f' *is the rate of fitness loss (the decay model). The probability of coalescence of two asexual nodes was proportional to population size of respective clone, until the time when the given clone was founded and all its variability coalesced into a single founder node, which was than considered as sexual. Any two sexual nodes could coalesce if they co-occurred in the same sexual deme with a probability proportional to its population size. For each set of parameter values, we performed 1000 independent simulation runs.

In each simulation run, we recorded the pedigree of fixed number of individuals per deme or per clone, respectively. The length of each branch was characterised by two numbers. First, we recorded total number of generations elapsed between its ancestral node and time of splitting into the daughter branches (or tip). Second, we recorded the time that each branch spent in an asexual state (this could be zero in case of joining two sexual nodes). Total number of *S *segregating sites was then overlaid onto each resulting pedigree of asexual individuals according to the infinite sites model as described in Pannell [59]. In order to evaluate the effect of genetic variability inherited from sexual ancestor, segregating sites were overlaid on branches either according to their total length (we call it 'total dataset'), or according to the number of generations they persisted in an asexual state only (we call it 'pruned dataset'). We then calculated the values of D and D* based on the mutation distribution in total and pruned datasets. The power of D and D* to detect the clonal decay was evaluated for each set of simulations as the proportions of simulated values lying outside the upper and lower 95% bounds of the neutral interval for given sample sizes [35,36].

### Material

In order to test for the evidence of clonal decay in real-world asexuals, we focused on complexes composed of polyphyletic assemblage of clones, each derived from one sexual species (or two species in case of hybrid asexuals), which may be approximated by our model of repeated recruitment of clones. We downloaded individual mitochondrial DNA sequences from GenBank according to original publications describing 15 asexual complexes (Table 1). Due to the low sample sizes of the other clades of *Timema*, we analysed only sequences from the Northern clade of Law and Crespi [11] labelled 'clade 4-2'). To test if such systems are not formed by independent asexual species, we followed a "4X rule" of Birky and Barraclough [16]. Briefly, phylogenetic tree was constructed from both sexual and asexual haplotypes to identify reciprocally monophyletic clusters. Mean pairwise sequence divergence (π) within each cluster was calculated as an estimate of Θ_π_. We finally checked whether the ratio of the sequence difference between clusters is less than a quadruple of the largest Θ_π_.

### Dataset preparation and pruning

We first prepared a dataset composed of entire sequences obtained from asexuals (the Total dataset). We also prepared a dataset composed of sequences from sexual individuals only. Subsequently, we prepared pruned datasets, which tend to eliminate the polymorphisms inherited from sexual ancestor. We propose below two approaches to achieve this goal. The first one is based on phylogenetic reconstruction, but because of low statistical support for some nodes in reconstructed pedigrees the second approach relies on different rationale and takes into account the distribution of polymorphic sites in the alignment:

a) We reconstructed the maximum parsimony (MP) tree from downloaded sequences using PAUP* 4.0b10 software [60] and mapped the transitions from sex to asexuality using the MP criterion. We assumed that only sex → asex transitions are possible. We recorded only mutations defining the relationships among asexual haplotypes and their connections to closest sexual nodes (this eliminated all mutations along branches characterised by sexual reproduction). For each individual we transformed all such mutations into binary state and overlaid on sequence of total length identical to original publication according to the infinite sites model. Since *Cobitis *gynogens stem from reciprocal crossings, we pooled their heterospecific mtDNA sequences into single dataset. We calculated D, D/Dmin and D* from such data.

b) The sequences of asexuals were aligned together with their sexual counterparts. All sites segregating in sexual progenitors were deleted from the alignment and "sexual" sequences were subsequently removed from the dataset. As such, we kept only polymorphic sites not found in sexuals, minimizing the confounding effects of sequence evolution during the sexual phase. Values of D, D/Dmin and D* were evaluated as above. In the cases of *Cobitis *and *Menetia *asexual datasets were separated into two sets of clonal lineages according to the source of their mtDNA due to the large number of polymorphic sites corresponding to divergence between parental species. We did not perform pruning of the dataset in the case of *Menetia *lizards since both asexual lineages were monophyletic with respect to their maternal sexual ancestors.

Both methods of pruning use different rationale and have different drawbacks. The former one assumes that all neutral mutations, which accumulated after the transition to asexual reproduction, occur according to an infinite-sites model. This seems reasonable in most complexes, where clones were only moderately divergent from nearest sexual ancestors. The latter method relaxes such an assumption, but it may eliminate positions, which mutated independently in sexual and asexual lineages. We note that both methods of pruning lead to very similar outcomes.

### Data analysis

DNAsp [61] was used to estimate Θ_π_, Θ_S_, Tajima's D and Fu and Li's D* from the aligned datasets as well as associated p-values by comparisons against 1000 coalescent simulations of neutral populations with the same number of segregating sites. Since the assessment of the heterogeneity among estimated index values may be affected by differences in number of polymorphic sites among datasets, we adopted the approach of Schaeffer [62] to account for possible bias. This method calculates the ratio of Tajima's D to its theoretical minimum value (Dmin) where all segregating sites are singletons with respect to the rare variant. This removes the dependence of Tajima's D on number of samples and segregating sites. The D/Dmin ratio will approach -1 as the rate of population expansion or selective sweep increases. All statistical analyses related to fitting of Linear Models were performed using R software [19].

## Competing interests

The authors declare that they have no competing interests.

## Authors' contributions

All authors collaborated on computer simulations and discussed the text. KJ analysed the sequence data and wrote the first draft of the manuscript. KJ and PD defined the main hypothesis and PD performed the statistical tests. All authors read and approved the final manuscript.

## Reviewers' comments

### Reviewer Report 1

Isa Schön

Royal Belgian Institute of Natural Sciences

Freshwater Biology

Vautierstraat 29

B-1000 Brussels

I have read this manuscript with great interest. By applying models from population genetics to asexuals, Janko et al. will most certainly bring new ammunition into the debate on the still unsolved paradox of sex. Their models provide further insights into the processes that may play a prominent role for the fate of asexual clones. It seems that there is some sort of age-dependent selection in the models and asexual systems that were used. I find this paper highly relevant and it most certainly deserves publication, even more so because it is controversial. Some of its methods are very innovative and relevant, also for future research and modelling on this topic. There are several issues, however, which need to be solved before it is ready to be accepted for publication. They are outlined below in different parts, following the structure of the manuscript. But I believe that most of these will be easily solved. In general, the manuscript would also greatly benefit from a thorough correction of the English throughout, preferably by a native speaker. In its current stage, it is full of grammatical mistakes and not always comprehensible, which is a great pity.

I. "Background" and "Does the distribution of asexual ages identify the nature and generality of underlying processes?"

Janko et al. criticize the paper by Neiman et al. heavily in the first part of their manuscript. I am no expert in statistics and can therefore not judge whether Neiman et al. used appropriate or wrong approaches. However, three reasons jump to my mind to explain the different results between the two author teams.

1. I find it a legitimate approach to use both young and old complexes as Neiman et al. did if one wants to test the general age distribution of asexuals. I also consider the redrawn Figure 1a in the current manuscript actually as support for the statement of Neiman et al.. The three ancient asexuals are still within the confidence intervals of this curve in Figure 1a, while the simulated asexuals fall outside of the confidence limits, at least in my view. Therefore, the description and discussion of these results need to be changed on pages 6 to 7.

The description of Figure 1b and the reasoning why there is a discrepancy between the two functions is unfortunately incomprehensible to me. I would urge the authors to either explain this much more extensively as any reader not being a statistic wizard will not be able to follow this part of the manuscript or drop it altogether. Likewise, I also find it necessary to illustrate the conclusions by Janko et al. on page 7 to 8 because readers being less familiar with statistics (as one would expect from a journal like Biology Direct) will again not be able to follow these discussions.

***Author's response: ****We have changed the Figure *1* as well as its description in order to avoid the confusion. We omitted confidence interval in Figure *1* to avoid misunderstanding. In fact, confidence intervals shown in original graph are used to estimate "mean response" (estimated Y) for given X. These intervals were calculated to show reliability of the fitted model and they cannot be used for estimation of observed values.*

*However, we can agree only partially with the first claim of Isa Schön. Indeed, to address the generality of underlying process, it may be a good idea to pool young and old complexes into single analysis. But as we demonstrate in the text, this problem may not be tackled by inspecting cumulative frequency distribution. Similar issue was clearly demonstrated decades ago by Preston in macroecology (please, refer to Results/Discussion, part 3.1). We applied Preston's approach to the case of asexuals and clearly show that observed cumulative frequency may be explained by simple model (as done in *[17]*) as well as equally-well (or even better) by more complex model taking into account several processes operating at different parts of the clonal age-spectrum. When the authors *[17]* used both young and ancient complexes to test for predictions of clonal turnover (this is our second critical remark), they have violated the basic assumptions of the tested model. Therefore, we agree that testing the general processes is possible from the dataset as in *[17]*, but it must be based on a model that takes into account both 'intraspecific' and 'interspecific' processes. This was not done by Neiman et al. *[17]* and this is the aspect, which we criticise.*

2. It might be a good idea to shorten the part of their manuscript where they criticize the manuscript by Neiman et al. further (p. 6 to 9) because most of the criticism can be explained by the mixed data set of Neiman et al. including both young and old asexuals (as Janko et al. say themselves). There might not really be a need here to go on about details on statistical "finesse" for the same reasons as stated above.

3. A third argument for my suggestion under point 2 is that it becomes obvious again in this manuscript that differences in the definitions of asexual species and clones might also be at play when regarding the different conclusions of Neiman et al. versus Janko et al. There have been ample discussions on this elsewhere (please also include Martens et al. 2009 on the question of asexual clone definitions), which could be cited, so that there is no need to elaborate too much on the rejection of the neutral model for distribution of clonal ages here.

In conclusion for the first part, it is in my opinion sufficient to outline the reasons shortly why Janko et al. do not follow the conclusions of Neiman et al. There is sufficient reason to go on with the new model approach while I actually find too much repetition on page 10 of results that are repeated again later. I therefore suggest shortening pages 6 to 10 except for the statistics of Figure 1b, which need to be extended.

***Author's response: ****We have shortened the first part by more than ¼ and we have reduced the statistical part to few points showing how Preston's critique is relevant to the analysis of Neiman et al. *[17]*. We also shortened the part elaborating on asexual species definition and use the concept of a clone discussed by Martens et al. *[32].

II. "Detecting the age-dependent selection against clones from sequence data".

I have some smaller comments on this part:

On page 11, it is not clear to me to which studies the authors refer when mentioning their predictions?

***Author's response: ****Dr. Timothy Barraclough criticised several parts of the MS, where our definitions of predictions were unclear. In the new version, we have avoided this problem; please refer below to our reply to Dr. Barraclough.*

On page 12:

Is there only migration from sexuals to asexual demes or also vice versa?

***Author's response: ****We assume only sex→asex migration. For more detail, please refer to Results/Discussion, part 3.1, 2^nd ^paragraph, and to our reply to A. Mushegian below.*

Is your model also monoclonal?

***Author's response: ****Our model assumed migration-drift equilibrium so in the case of low recruitment rate of clones, it would eventually lead to monoclonality. Therefore, it is also applicable to monoclonal systems.*

I would like to see the per-genome deleterious mutation rate U.

***Author's response: ****The effect of deleterious mutation rate is now demonstrated on Figure *3a.

The statement that the age-dependent loss of fitness is a monotonous function

of f' is only true for certain m but not for all.

***Author's response: ****We are convinced that our text is correct here: indeed, migration among sexual demes (m), had no effect on the pruned dataset and neutrality indices decrease monotonically with increasing f'*

On page 13:

Which index values are increased (top sentence)? Are these values of genetic neutrality?

***Author's response: ****Yes, we meant the neutrality indices and we made the text clearer.*

I would like to see somewhere the evidence that genealogies spend relatively longer time in structured sexual population. This is not obvious to me from Figure 5.

***Author's response: ****We have modified the Figure *5* to make this clear now. Please see the Figure *5* and its legend.*

Likewise, it would be nice to be able to see somewhere that population structure and low rates of clonal recruitment soothe clonal decay and lead to longer internal branches in asexual pedigrees. I consider these important results from the modeling, which should be made more accessible to readers. The last sentence of this part needs more explanation - again, this is an important result but it is dealt with a bit too briefly.

***Author's response: ****We have modified the text about this issue to make it clearer. However, we do not include a figure demonstrating it because it is shown in both cited papers (*[13], [33]*) and we have already rather large amount of figures. Both cited studies demonstrated that population structuring attenuates clonal decay and leads to longer-than-expected internal branches in genealogies (positive values of neutrality indices). Population fluctuations do the opposite as follows from the coalescent theory (e.g. *[37]*). Therefore, we believe that verbal discussion with appropriate references is adequate for our purposes.*

III. "Testing for clonal decay in real populations."

I agree on the whole with the method of pruning although it is probably only valuable if the variance inherited from sexual ancestors is higher than the neutral and deleterious mutation rates together. This should be mentioned somewhere on page 14.

***Author's response: ****We agree but we have to add that pruning of the data should always remove the 'noise' from inherited polymorphisms. Of course, if such inherited polymorphism constitutes large portion of total variability, the 'noise' would be more prominent. We have added this to the text; please see Results/Discussion, part 3.1.*

It is a very rough estimate indeed to use body weight and body length as proxies for population sizes. Also small animals can be really rare. Would it not be possible to include some real data on this? I find it hard to believe that these are not available at all for the taxa included. This would allow investigating the important effects of population size in a more appropriate fashion, I believe that the way population size was estimated, is also the reason why there was such a bad model fit (see below).

***Author's response: ****We were really unable to find any sound data on population sizes. For more discussion about this issue, please see below our reply to Timothy Barraclough.*

I find that predictors 3, 4 and 5 are somehow related - extreme habitats are often in geographic isolation or at different latitude, especially in patterns of geographic parthenogenesis. I therefore suggest that the authors add this.

***Author's response: ****We agree and we have modified the text accordingly, please see Results/Discussion, part 3.1, predictor 4 as well as the Table *2.

I do not believe that the number of available data on asexual complexes is low. It rather seems true that the number of data fulfilling the criteria of the authors is low - this should be clarified on page 17.

***Author's response: ****We have removed the sentence about the need to use collect more data. However, we still believe that the amount of well-studied asexual complexes is rather low. Please note that other attempts of meta-analytical approaches (*[17], [15]*) did not use much more available datasets than we did. But we agree that only a fraction of such datasets is suitable for our test.*

The description of R2 values for the different variables from Table 2 needs to be corrected: R2 for D* in Pruning 1, for example, is 0.3601 and thus lies not between 0.64 and 0.43. Similarly, it is correct that data pruning and sampling effort (models 7 &8) have a poor fit, but so does model 9 (body length), where the fit is worse in D/Dmin of Pruning 1 and 2 than for models 7 and 8.

***Author's response: ****We have corrected the text; please see Results/Discussion, part 3.2.*

I might have overlooked something, but is not clear to me why you would expect that index values for sexual species should significantly correlate with their latitudinal distribution (p. 18). Please provide some additional explanation on this somewhere.

***Author's response: ****It follows from the fact that areas in higher latitudes are more prone to founder-flush events related to glacial cycles. Such events lead to negative index values. We have extended the description of this issue; please see Results/Discussion, part 3.1., predictor 4 and part 3.2. paragraph 3.*

"Conclusions"

It is very much appreciated that the authors are critical of their own modeling results (p. 19). The question how much models reflect the real world, is a very important one. I find the way that the asexual systems were selected to be included in the models at the same time a major strength and weakness of this manuscript. I agree with the authors that mechanisms might be very different in young, asexual systems that are still closely related (both in time and space) to their sexual roots and ancient asexuals. By only including one kind of asexuals, a major weakness of the models might be excluded and the results might be more genuine. However, on the other hand, this approach excludes older systems with probably different mechanisms but which are equally interesting. I would suggest that the authors add wherever they describe and discuss their major result, namely that age of clonal complexes seems to be the most important variable that this is only true for the young systems that were studied. It might be very different for ancient asexuals, mixed systems with different species, etc.

In this respect, it would be good to add other possibilities to the future suggestions for models such as the ones mentioned above.

***Author's response: ****We agree that our type of analysis is suitable only for asexual complexes, where each new clone enters the same arena for selection and drift with all other clones. This may be limiting, since our analysis does not test processes in ancient clonals. On the other hand, Isa Schön just mentioned that mechanisms might differ between young and old asexuals. In fact, exactly this argument is a valid critique of previous approach by Neiman et al. *[17]*. They suggested that most parsimonious explanation of observed age distribution is that mechanisms are the same in all asexuals. We have showed that such mechanisms are likely to differ. We believe that we are very explicit about this in the text.*

I wonder to which extent and how cyclic parthenogenesis and egg banks as they occur in Daphnia, which was included in the analyses, could affect your general outcome of increasing strength of age-dependent selection in older clones. This could be another idea for future models?

***Author's response: ****As far as we know, studied populations of Daphnia that are included in our analysis are obligate parthenogens (see *[65]*). Therefore, it should not affect our analysis.*

When discussing selection (such as on page 21), it would very much enrich the discussion if the authors could include and relate to other concepts of selective response of clones, such as e.g. the Frozen Niche concept and General Purpose Genotypes.

***Author's response: ****At this stage we are not sure, how our results relate to such concepts. We indeed showed that signs of selection become stronger in complexes with older clones. In this sense, our analysis yields some proof to the hypotheses of age-dependent selection against clones. However, such selection is not inherent in Frozen Niche or General Purpose Genotype hypotheses. Therefore, we didn't find a simple way how to extend our discussion with regard to those concepts.*

There is some contradiction - whereas it is said in the first paragraph on page 21 that analytical solutions for polyclonal complexes are still missing, it is said in the conclusions that the study indicates the suitability of sequence data to address selective forces, even in polyclonal complexes. This needs to be resolved.

***Author's response: ****We suggest that analytical solutions obtained by other studies of monoclonal models are not simply applicable to polyclonal systems. On the other hand, we cited works that analytically tractate equilibria in metapopulations, which gives us some hope that in the near future; such analytical tolls shall be available even for polyclonal systems. Our study provides no analytical solution, since we used individual-based and coalescence simulations.*

There are many phylogeographic data available already from asexuals, including plants and a lot of literature on geographic parthenogenesis; my feeling is that these have not been included here because of the rather strict criteria that were applied. Therefore, the call for phylogeographic studies needs to be rephrased.

***Author's response: ****We have rephrased it; please see our reply to similar remark of Isa Schön above.*

"Methods"

I am not certain whether finite linear stepping stone models do fit migration patterns of the investigated taxa. This needs to be addressed when discussing the results. Especially in aquatic asexuals, many taxa have dry-resistant eggs, which are distributed easily by wind or birds and thus violate the stepping stone model.

***Author's response: ****Our model was not intended to address all specific aspects of dispersal (in fact, this would be very difficult given the broad taxonomic spectre of studied organisms). Our aim was to study the impact of population structure on genetic variability of asexuals. For that purpose, it is sufficient to demonstrate that increasing fragmentation results in higher index values.*

It would be nice to see how the segregating sites were overlaid onto each pedigree of asexuals. Maybe, this could be shown in supplementary material?

***Author's response: ****We used standard approach, when fixed number of segregating sites is overlaid onto simulated pedigree. The number of mutations overlaid onto given branch of the pedigree is proportional to its length. This has been described in detail in other works, e.g. in *[59].

As said above, models are only as good as the examples to which they are applied. I am aware of the reasons why the authors applied their criteria and to which selection that led, but I still have some comments which I feel should be discussed, especially if the results are described as general as they are now. My comments might indicate that the outcomes might differ if these criteria would be taken into account:

1. polyphyletic origin, hybrids: most of this will lead to polyploids, which are known to suffer less from the predicted accumulation of mutations and also from effects of Muller's ratchet. I would therefore like to know which examples were polyploids and would like this to either be tested or at least discussed.

***Author's response: ****This is inspiring remark. As suggested, we reanalysed the data and included the test of the effect of polyploidy (please, see Results/Discussion, part 3.1, predictor 7). This partitioning did not explain the data to a large extent, suggesting that polyploidy does not have significant effect on genetic variability of neutral loci in asexuals.*

2. I have some reservations about using different sources of sequence data - the sequences were, as is said, "mostly mitochondrial" - there are huge differences between nuclear and mitochondrial genomes, also regarding mutation rates. I agree that the two genomes should be linked in asexuals but since we are dealing with very young systems, I would like this to be considered somewhere. It might be more appropriate to only use mtDNA data.

***Author's response: ****We expressed ourselves wrongly. In fact, all data are mitochondrial. We made it clear in this version.*

3. Which method of Birky and Barraclough was used to test for asexual species and how?

***Author's response: ****We have included the detailed description to the Methods.*

4. A mutation rate of 0.5 if very high - how are the results with lower mutation rates or was this not tested? On which bases was this mutation rate selected?

***Author's response: ****Actually, this mutation rate is not unrealistic. This rate is per-genome. Rates around 1 mutation per generation are often discussed in theoretical studies on Muller's ratchet. Gordo et al. *[30]* used a range between U = 0.01 and U = 0.1 when studying the evolution of sex chromosome (i.e. per-chromosome mutation rate). Therefore, we consider our choice as appropriate. To address this concern, we have also simulated different values, ranging from U = 0.05 to U = 0.5 as shown on Figure *3.

Tables:

Some abbreviations should be explained in Table 1. What are LGM, SAS, SAR, RP and WP?

***Author's response: ****We have added the explanations to Table *1.

Figures:

I did not receive Figure 7, so I cannot check what was described there (for example, on page 18).

***Author's response: ****We are sorry for this inconvenience. The figure must have been lost during E-mail transmission since we have included it to the original message. Once again, sorry for this.*

### Reviewer Report 2

Arcady Mushegian

Director of Bioinformatics Center

Stowers Institute for Medical Research

Kansas City, Missouri 64110, USA

The manuscript by Janko et al. examines the question of the "clonal decay", i.e., the explanation of the recent age of asexual, clonal populations relative to their sexually reproducing relatives by invoking strong selection against the former, resulting in a rapid purge of asexual lineages, so that only those that have recently become asexual are observed. The authors have put forward an alternative explanation for these observations, based on lineage birth-and-death model.

The first part of the study is the review of the evidence; the authors' suggest that most of it is explained by stochastic birth-and-death effects as well as, if not better than, by negative selection models. They then go on to show more of the same by simulating both processes in silico.

Finally, the authors suggest several tests to distinguish between intense selection against clonal lineages and their rapid turnover. It is this part that becomes confusing. If I understand the results correctly, they in effect say that there is no evidence that exactly one of these factors is in play, and that it is more likely that both are. If this is indeed a conclusion, then perhaps the whole paper should be rewritten with the emphasis on the two hypothesis not being mutually exclusive.

One other system where clonal populations are also thought to experience rapid lineage turnover, and where at the same time selection can be measured more directly, are viruses. There are no sexual populations from which viruses are constantly derived, but I wonder whether the authors have given any thought to this.

On p. 25, the authors assumed that only sex→asex transitions are possible (i.e., the base observed in the sexual species is ancestral, and the base observed in a sister asexual clade is derived). This should not be assumed: use an outhgroup to determine the ancestral state, and retain only those sites at which this state can be so determined. The results may be the same or almost the same as with the authors' assumption, but there is no reason not to do it.

*Author's response:*

We agree that main conclusion was a bit hidden in the text of the first version of the paper. We changed relevant parts of the manuscript with special care to show that:

a) neutral process (clonal turnover) is a special case of the model with age-dependent selection against clones (clonal decay) - please, see the Background, 4^th ^paragraph.

b) Both models give quantitatively similar predictions and their parameterisation is difficult. However, relative importance of distinct parameters is expected to correlate with different biological traits. Hence, we performed a comparative analysis to find a trait with highest explanatory power - please, see Results/Discussion, part 3.1.

c) The distribution of observed data notably differs from predictions of both models, which imply some age-dependent selection against clones and suggest that genetic diversity of natural asexual complexes may not be explained by neutral processes alone. However, as one of the main messages, it suggests that proximate mechanism of such selection is different from simple deleterious mutation accumulations, which was used in many previous studies - please, see the Results/Discussion, part 3.2, 4^th ^paragraph.

We have considered a possibility to extend our model to viruses. However, as also recognised by Arcady Mushegian, we found no clear parallel between the model of asexual complex and viruses. This is because viruses do not recruit from sexual population. More or less frequent recruitment of new clones is the core of our model. Such model therefore rather approximates mixed sexual and asexual populations than systems, where sexuals do not exist. This reasoning is also mentioned in Results/Discussion, part 1, 5^th ^paragraph and part 3.1, 2^nd ^paragraph.

Finally, we agree that incorporation of bi-directional gene flow between asex and sex would relax one strong assumption of our model. Indeed, it may be possible through parsimony mapping and resulting polarisation of mutations on the phylo tree may be different than in our approach. However, we decided not to do this for several reasons, which are explained in the text with appropriate references (please, see also the Results/Discussion, part 3.1, 2^nd ^paragraph). Shortly, in many complexes (especially in the cases, where strict parthenogenesis has been experimentally demonstrated and in case of hybrid asexuals) asex → sex transitions are very, very unlikely. Moreover, in other asexuals, where occasional gene flow into sexual population is mediated via males mating with sexual females, the marker used in this study - mtDNA - would not be affected. mtDNA remains tightly bound to clones, once 'frozen' in clonal lineage. Therefore, parsimony mapping on phylo trees would have to assume unequal probability of transitions in both direction and at the moment, we do not see, how to weight it. For those reasons, we believe that the simplifying assumption of zero asex→sex transitions is justified.

### Reviewer Report 3

Prof. Timothy G. Barraclough,

Professor of Evolutionary Biology

Division of Biology,

Imperial College London,

Silwood Park Campus,

Ascot, Berkshire, SL5 7PY, UK

E-mail: t.barraclough@imperial.ac.uk

This paper addresses the interesting question of whether the theorized decline in fitness through time of asexual populations (called clonal decay) can explain the observation that most asexuals tend to have originated recently from sexual ancestors. In a previous paper, which forms the starting point for the present study, Janko and colleagues made an important advance in this field - patterns that look like the outcome of clonal decay might in fact be explained by neutral turnover of asexuals. The present paper falls into two parts: a discussion and critique of some other recent papers on this issue; and an analysis combining modelling with a comparative survey of genetic signatures and possible covariates across a set of asexual taxa and their sexual progenitors.

The discussion of previous papers is potentially useful and critical, although in parts it assumes greater familiarity with the previous work than I have. The second part has noble aims, and at the very general level of linking theory with comparative population genetics is to be strongly applauded. I agree whole-heartedly that comparative population genetics is an extremely promising but relatively neglected avenue for study. Unfortunately, I do not think the analyses shed any real light on the proposed questions, for the following reasons:

1) A very general point - the focus on the phenomenon of clonal decay does not seem especially helpful to me. This term appears not to be of widespread usage in the literature, and as defined here encapsulates two mechanisms: both the accumulation of deleterious mutations and the failure of asexuals to adapt quickly enough to keep pace with changing environments. To my mind, the use of a phenomenological definition rather than a mechanistic one does not help in getting to grips with the causes of observed patterns.

2) Somewhere early on it needs to be stated that the clonal turnover model assumes unidirectional shifts between sexuals and asexuals, otherwise there would be no reason to expect sexuals to be ancestral at equilibrium. Even so, my intuition is that the observed pattern of recent asexuals across animals and plants would be hard to explain solely by random but biased turnover - what stops asexuals replacing the sexual ancestor? In the present model, it seems to be the assumption that sexuals and asexuals belong to separate populations/demes that are independently limited. What is the biological basis for this assumption? Of course this is a matter for quantitative evaluation, which is the aim of this study, but I'm left feeling that there are important assumptions behind the model that aren't fully explained in the manuscript.

3) The predictions of the clonal turnover versus clonal decay models are not clearly explained. In many parts, predictions are stated without sufficient explanation and without stating briefly the assumptions behind the prediction. Examples include: "this was interpreted as evidence against clonal turnover, which predicts negative correlation between clonal diversity and age"; "allopatric populations should possess older clones"; "predictions of Higgs and Woodcock hold even in asexual complex of multiple clones"; "clonal ages should be a strong predictor of observed data distribution and the correlation should be negative". Some of these statements might be justified in the earlier papers, but they need to be explained well enough in the present paper to be understandable without referring to earlier work.

4) My main problem is that I do not believe that the proposed genetic signatures are useful tools for disentangling the forces of interest here. There are multiple demographic and selective effects that influence the value of signatures such as Tajima's D. For example, a recent selective sweep within an obligate asexual population would lead to a negative D, potentially even if there were no significant deterioration in fitness over time. Population expansion would have a similar effect. This problem is acknowledged, but the proposed approach of comparative analysis of covariates of observed values constitutes very weak inference, in my view, for the following reasons.

i) Even if the predictions are correct for the neutral turnover model, it is not clear that other models would not generate similar predictions. I imagine that a large suite of models could predict that Tajima's D would correlate with clone age.

ii) The actual statistical tests rely on surrogates for underlying values of interest. For example, prediction 2 concerns population size, but body weight is used as a surrogate for population size. Latitudinal distribution features in predictor 4 and 5, but there are many well-known covariates of latitude. The combined effects of large, unquantified errors in the use of surrogates, and the possibility of other confounding factors not considered here, means these tests offer negligible power to distinguish alternative predictions (which themselves seem to have an ill-defined basis).

iii) These problems seem borne out by the results, which contradict predictions, although the details are hard to follow (see next point). Most covariates yield no explanatory power, and there is no validation that they accurately reflect the underlying variables of interest. So, null results can only be interpreted as insufficient power.

5) The presentation of results and their interpretation are very hard to follow. It is stated that a "negative correlation between neutrality indices and clonal ages, ...was not the case (Figure 7)" but Figure 7 shows a negative relationship. Under predictor 1, it says the correlation should be negative, because "younger clones should display more negative indices" - surely this is a positive relationship. The interpretation of this main result is even harder to follow: the result is discussed as indicating merit for the approach, even though it is in the opposite direction to predicted. Given all the problems concerning the likely low power of these tests, it is impossible to draw any conclusions from this exercise.

To conclude, I believe a comparative study of signatures of selection across a suite of asexuals and their progenitor sexuals, guided by theoretical models, is an extremely worthwhile goal. However, the limitations of the present study prevent any clear conclusions being reached, in my opinion.

*Author's response:*

We really appreciate that Timothy Barraclough wrote and signed the review of our manuscript, which he does not agree with. We have to apologise for some confusion related to points 2, 3 and 5 of his critique.

ad 2) We agree that model assumptions should have been described with greater detail rather than just cite our previous work. We have extended the relevant parts of the text. The discussion about biological relevance of sex→asex transitions may also be found in our reply to Arcady Mushegian.

ad 3) We have extended relevant parts of the MS by short explanation of each model prediction and coupled it with appropriate references.

ad 5) We wrongly wrote in the original version that predicted correlation between clonal age and neutrality indices is negative. We had in mind that intensity of clonal turnover/decay negatively affects the life span of clones and drives neutrality indices into negative values. Indeed, resulting correlation should be positive as correctly stated by Dr. Barraclough. We are sorry for this confusion and we have corrected the text accordingly.

However, our opinion differs from that of Timothy Barraclough in other points and especially his fourth remark requires more detailed explanation. Let us address Timothy Barraclough's criticism point by point:

*ad 1) Indeed, the term 'clonal decay' is not widely used in the literature. However, given the aim of our study, we had to use some term for a class of mechanisms responsible for age-dependent fitness decrease of clones. The term 'asexual decay' is used in literature dealing with evolution of sex chromosomes (e.g. Schartl 2004. Current Opinion in genetics & development 14: 634-631). Since we are dealing with processes summarized over individual clones, we found 'clonal decay' suitable. It is simple and was used in our previous paper as well as in *[17]*. We defined it in the Introduction and hence, we hope that its usage simplifies the text. Using alternative and longer terms such as 'mechanisms of long-term disadvantage of asexuality', or 'age-dependent selection against clones' would not be practical. We do not insist on keeping this term in the text, but we found it useful.*

*ad 2) Timothy Barraclough is right that simple neutral turnover of clones cannot explain the persistence of sex in the nature. However, we never claimed it could. Instead, it may well explain observed age-distribution of clones, which was proven in our previous paper *[13]*. So far, quite a lot of different explanations have been proposed for the persistence of sex, some implying long-term advantages and some short-term advantages. Given that the focus of our study is to detect the footprints of age-dependent selection against clones from the genetic variability (we do not study why sex persists), it seems instructive to us to narrow the parameter space of models.*

We agree that our model is a simplification by assuming independent limitations of sexual and asexual populations. But most useful models are. Modelling the interactions between sex and asex in the whole complexity should include the effects of plethora of parameters, which would be numerically almost intractable. We are not aware of any study that would have as ambitious aims as to model the whole complexity of sex-asex interactions. Instead, other studies investigated just one or few such processes (e.g. Peck et al. 1998. Nature 391: 889-892; Gordo and Charlesworth, 2000. Genetics 154: 1379-1387, Doncaster, Pound, Cox 2000. Nature 404:281-285). Moreover, our assumption has a biological background. Many asexual complexes are composed of hybridising sexual species and their asexual polyploid hybrids. Hybrid and polyploid forms are often assumed to differ from parental diploids in many traits (e.g. Stillwell and Benfey. 1995. Aquaculture 137, 355-358). Such difference may attenuate the competition and minimize the niche overlap between sexual and asexual forms. Therefore, we believe that our model, as constructed, provides novel insights and allows us to focus on the effect of studied parameters, i.e. effect of clonal decay and turnover on the shape of genealogy of asexual individuals. We do not suggest that there are no mechanisms counterbalancing the two-fold advantage of asexuals. To the contrary, we are convinced that there must be some. However, in this paper we showed that

a) previously used arguments about long-term disadvantage of asexuals are inconclusive (Introduction and Results/Discussion, part 1);

b) sequence variability of asexuals is likely to bear traces of selective process affecting them (Results/Discussion, part 2);

c) mechanisms underlying the age-dependent selection seem to exist but their nature differs from assumptions of available models and it is not yet understood (Results/Discussion, parts 3.2 and 3.3).

ad 4) Indeed, various demographic or selective processes may affect asexual pedigrees but we have taken many of them into account explicitly. We must also stress that such processes would affect Tajima's D and clonal ages in a predictable manner and our simulations show that population fragmentation and fluctuation will cause positive correlation of both such parameters, which contrasts natural observations (this was mentioned in the text and we put more emphasis on it in the reviewed version, please see Results/Discussion, parts 3.1 and 3.2).

Timothy Barraclough further suggests that selective sweeps of individual clones would cause negative values of neutrality indices even if there were no significant deterioration in fitness over time. We agree. However, the sweep of any new clone at the expense of older ones would also result in younger mean age of clones in the whole asexual complex. Again, as predicted by our model, we would end-up with positive correlation between neutrality indices and clonal ages. Moreover, if such process happens rarely, our model would not be largely sensitive to it since the pruned dataset takes into account polymorphisms from the whole polyclonal population (see Results/Discussion, part 3.2, 3^rd ^paragraph). On the other hand, if sweeps of young clones happen frequently, would it still be justified to assume that 'there were no significant deterioration in fitness over time'?

Our predictions would only be contradicted by sweeps of old clones at the expense of younger ones. We have to stress that such events are not taken into account by traditional hypotheses assuming fitness deterioration of old clones either. Therefore it does not disqualify our study, which addresses the power of population models to study the effects of neutral and selective processes on clonal genealogies. We have included short discussion of this issue into the text; please see Results/Discussion, part 3.3, last paragraph.

*ad 4i) We fully agree that some underlying mechanisms must exist to explain observed significant negative correlation between neutrality indices and clonal age (see Figure *8*). However, such a fact does not disqualify our study, since we are the first ones to point at this important correlation, which help explain the nature of underlying mechanisms. We have devoted two pages to the discussion about this issue. In the new version of the text, we summarize such discussion in the point 3.3. The main message of our paper is that contemporary models of selection against clones are insufficient to explain the underlying mechanisms.*

ad 4ii and 4iii) We do not agree in this point. As we showed in Results/Discussion, part 3.1, linkage of whole clonal genomes prevents standard multilocus-based tests of selection and indirect tests are necessary. Let us also remind that used surrogates have quite sound background:

a) Latitudinal distribution proved to have very strong effect on sexuals, which is consistent with hypothesis of founder-flush events (see Results/Discussion, part 3.1, predictor 4). Our study clearly shows that asexuals are under control of quite different mechanisms than their sexual ancestors since asexual's genetic variability is not affected by latitude (see Results/Discussion, part 3.2.).

b) Isolation from sexual population significantly affects the distribution of clonal ages, which agrees with the predictions of neutral model. It is therefore valid and useful approach to study its effect on sequence variability (see Results/Discussion, part 3.1, predictor 3).

*c) Usage of body size as a proxy for population size was adopted from previous literature on species with unknown population size. We have newly added an indication that larger sexual species (with putatively smaller population size) tend to have lower genetic polymorphism (although this trend was not significant; see Results/Discussion, part 3.1, predictor 2; Figure *6*). We therefore consider it as valid approach. Moreover, we did not reject the standard model of Muller's Ratchet solely from fitting the body size into the model. We only used it as supportive argument for our statement that standard models of clonal decay do not explain the data well (note that Muller's Ratchet predicts positive correlation between neutrality indices and clonal ages, while we observed the opposite)*

In a summary: we are convinced that application of all surrogates is justified on sound basis. Some of them significantly and predictably explain the variability of sexuals, others explain the distribution of clonal ages and yet others have been used in agreement with previous studies. Therefore, we may not agree with Timothy Barraclough that null results only suggest low power of the test.

Finally, in contrast to Timothy Barraclough, we do think that our study shed light on proposed questions. We have clearly pointed on problematic application of previous studies addressing the clonal decay. We have further demonstrated that deviations from neutrality increase with increasing age of clones and we also have proposed how to adapt future population model to treat this issue (please see Results/Discussion, part 3.3).
